# Anthropometric measures and arsenic methylation among pregnant women in rural northern Bangladesh

**DOI:** 10.1016/j.envres.2023.116453

**Published:** 2023-10-01

**Authors:** Tyler J.S. Smith, Ana Navas-Acien, Sarah Baker, Caryn Kok, Kate Kruczynski, Lindsay N. Avolio, Nora Pisanic, Pranay R. Randad, Rebecca C. Fry, Walter Goessler, Alexander van Geen, Jessie P. Buckley, Md Hafizur Rahman, Hasmot Ali, Rezwanul Haque, Saijuddin Shaikh, Towfida J. Siddiqua, Kerry Schulze, Keith P. West, Alain B. Labrique, Christopher D. Heaney

**Affiliations:** a Department of Environmental Health & Engineering, Johns Hopkins Bloomberg School of Public Health, Baltimore, MD, USA; b Department of Environmental Health Sciences, Columbia University Mailman School of Public Health, New York, NY, USA; c Department of International Health, Johns Hopkins Bloomberg School of Public Health, Baltimore, MD, USA; d Department of Environmental Sciences & Engineering, University of North Carolina at Chapel Hill Gillings School of Global Public Health, Chapel Hill, NC, USA; e Institute of Chemistry – Analytical Chemistry, University of Graz, Graz, Austria; f Lamont-Doherty Earth Observatory, Columbia University, Palisades, NY, USA; g Department of Epidemiology, Johns Hopkins Bloomberg School of Public Health, Baltimore, MD, USA; h JiVitA Maternal and Child Health and Nutrition Research Project, Rangpur, Bangladesh

**Keywords:** Adiposity, Anthropometry, Arsenic metabolism, Micronutrient status, One-carbon metabolism, Pregnancy

## Abstract

**Introduction:**

Arsenic methylation converts inorganic arsenic (iAs) to monomethyl (MMA) and dimethyl (DMA) arsenic compounds. Body mass index (BMI) has been positively associated with arsenic methylation efficiency (higher DMA%) in adults, but evidence in pregnancy is inconsistent. We estimated associations between anthropometric measures and arsenic methylation among pregnant women in rural northern Bangladesh.

**Methods:**

We enrolled pregnant women (n = 784) (median [IQR] gestational week: 14 [13, 15]) in Gaibandha District, Bangladesh from 2018 to 2019. Anthropometric measures were BMI, subscapular and triceps skinfold thicknesses, and mid-upper arm circumference (MUAC), fat area (MUAFA), and muscle area (MUAMA). Arsenic methylation measures were urinary iAs, MMA, and DMA divided by their sum and multiplied by 100 (iAs%, MMA%, and DMA%), primary methylation index (MMA/iAs; PMI), and secondary methylation index (DMA/MMA; SMI). In complete cases (n = 765 [97.6%]), we fitted linear, beta, and Dirichlet regression models to estimate cross-sectional differences in iAs%, MMA%, DMA%, PMI, and SMI per IQR-unit difference in each anthropometric measure, adjusting for drinking water arsenic, age, gestational age, education, living standards index, and plasma folate, vitamin B12, and homocysteine.

**Results:**

Median (IQR) BMI, subscapular skinfold thickness, triceps skinfold thickness, MUAC, MUAFA, and MUAMA were 21.5 (19.4, 23.8) kg/m^2^, 17.9 (13.2, 24.2) mm, 14.2 (10.2, 18.7) mm, 25.9 (23.8, 28.0) cm, 15.3 (10.5, 20.3) cm^2^, and 29.9 (25.6, 34.2) cm^2^, respectively. Median (IQR) iAs%, MMA%, DMA%, PMI, and SMI were 12.0 (9.3, 15.2)%, 6.6 (5.3, 8.3)%, 81.0 (77.1, 84.6)%, 0.6 (0.4, 0.7), and 12.2 (9.3, 15.7), respectively. In both unadjusted and adjusted linear models, all anthropometric measures were negatively associated with iAs%, MMA%, and PMI and positively associated with DMA% and SMI. For example, fully adjusted mean differences (95% CI) in DMA% per IQR-unit difference in BMI, subscapular skinfolds thickness, triceps skinfold thickness, MUAC, MUAFA, and MUAMA were 1.72 (1.16, 2.28), 1.58 (0.95, 2.21), 1.74 (1.11, 2.37), 1.45 (0.85, 2.06), 1.70 (1.08, 2.31), and 0.70 (0.13, 1.27) pp, respectively.

**Conclusions:**

Anthropometric measures were positively associated with arsenic methylation efficiency among pregnant women in the early second trimester.

## Highlights

• Anthropometric measures were positively associated with arsenic methylation efficiency in pregnancy.
• Associations were independent of plasma biomarkers of micronutrient status.
• Results suggest adiposity was more relevant than lean mass.

## 1 Introduction

Globally, an estimated 94–220 million people are exposed to drinking water arsenic above the World Health Organization's standard of 10 μg/L (Podgorski and Berg, 2020). Arsenic exposure causes bladder, lung, and skin cancers (IARC, 2012) and has been associated with cardiovascular disease and diabetes mellitus (Grau-Perez et al., 2017; Moon et al., 2017; Navas-Acien et al., 2019). Arsenic exposure in pregnancy has been associated with maternal morbidity (Kile et al., 2014), adverse pregnancy outcomes (Ahmed et al., 2019), impaired neurodevelopment (Vahter et al., 2020; Valeri et al., 2017), altered immune responses (Attreed et al., 2017; Farzan et al., 2016; Heaney et al., 2015; Raqib et al., 2017; Welch et al., 2019), and decreased lung function (Ahmed et al., 2017; Dauphiné et al., 2011).

An important modifier of arsenic toxicity is arsenic methylation (Kuo et al., 2017). After ingestion, inorganic arsenic (iAs) is methylated to form monomethyl arsenic (MMA) and dimethyl arsenic (DMA) compounds (Vahter, 2002). MMA and DMA are excreted in the urine together with iAs (Vahter, 2002). Arsenic methylation is commonly assessed by the proportions of arsenic species excreted in urine relative to their sum (iAs%, MMA%, DMA%) or by ratios of these species such as the primarily methylation index (MMA/iAs; PMI) and the secondary methylation index (DMA/MMA; SMI) (Kuo et al., 2017). In general, arsenic methylation efficiency refers to higher DMA% and SMI. The role of arsenic methylation in relationships between arsenic exposure and disease appears to vary by outcome. In non-pregnant adults, lower arsenic methylation efficiency has been associated with cancer and cardiovascular disease while higher efficiency has been associated with diabetes mellitus (Kuo et al., 2017). A small number of studies suggest that higher arsenic methylation efficiency in pregnancy may protect the fetus and offspring against some adverse outcomes. Maternal DMA%, for example, has been positively associated with placental and birth weight in Mexico (Laine et al., 2015). Maternal SMI has been positively associated with children's cognitive development and found to attenuate inverse associations between maternal arsenic exposure and children's lung function in the United States (Signes-Pastor et al., 2021, 2022). While reducing arsenic exposure remains the priority, a better understanding of arsenic methylation in pregnancy may help to identify additional public health interventions.

In several studies, body mass index (BMI) has been negatively associated with iAs% and MMA% and positively associated with DMA% in non-pregnant adults (Abuawad et al., 2021; Bommarito et al., 2019; Gamboa-Loira et al., 2021; Gomez-Rubio et al., 2011; Gribble et al., 2013) While a few studies in non-pregnant adults have been less consistent (Huang et al., 2007; Lindberg et al., 2008), results in pregnant women have been especially discordant. In Spain, BMI measured before pregnancy was negatively associated with iAs% and MMA% and positively associated with DMA% measured in the late first or early second trimester (Soler-Blasco et al., 2021). In Bangladesh, however, BMI measured in the first trimester was not associated with arsenic methylation measured at the same time (Li et al., 2008). In a second study in Bangladesh, BMI measured in the first or early second trimester was negatively associated with MMA% but not associated with other arsenic methylation measures measured at the same time, and BMI in the first or early second trimester was not associated with any arsenic methylation measure in the third trimester (Gao et al., 2019). Pregnancy is accompanied by increases in lean and fat mass reflected by BMI and by increases in arsenic methylation efficiency reflected by lower iAs% and MMA% and higher DMA% (Concha et al., 1998). Thus, relationships between BMI and arsenic methylation may vary between non-pregnant adults and pregnant women, and within pregnancy.

Additionally, two limitations of existing data in pregnancy should be addressed. First, as noted, BMI reflects both lean mass (*e.g.*, muscle, bone) and fat mass (Stevens et al., 2008). By considering several measures, such as BMI, skinfold thicknesses, mid-upper arm circumference, mid-upper arm fat area, and mid-upper arm muscle area, we might better understand why associations occur. Second, arsenic methylation depends on the availability of micronutrients involved in one-carbon metabolism, such as folate and vitamin B12 (Abuawad et al., 2023; Bozack et al., 2019). If anthropometric measures and micronutrient status are correlated, associations between anthropometric measures and arsenic methylation may be confounded by micronutrient status. While associations between BMI and arsenic methylation in pregnant women have persisted after adjustment for micronutrient intake estimated with food frequency questionnaires (Gao et al., 2019; Soler-Blasco et al., 2021), intake does not account for changing physiological requirements in pregnancy (Bae et al., 2015). Micronutrient status can worsen as pregnancy advances and the growing fetus draws on maternal stores (Velzing-Aarts et al., 2005). Adjustment for biomarkers of micronutrient status, such as plasma folate, vitamin B12, and homocysteine, may better control for potential confounding. Our objective was to estimate associations between multiple anthropometric measures and arsenic methylation among pregnant women in the early second trimester in rural northern Bangladesh, adjusting for micronutrient status and other confounders. We hypothesized that anthropometric measures would be positively associated with arsenic methylation efficiency but that associations would partially attenuate with adjustment for micronutrient status.

## 2 Materials and methods

### 2.1 Study design and sample

The Pregnancy, Arsenic, and Immune Response (PAIR) Study is a prospective pregnancy and birth cohort based at the JiVitA Maternal and Child Health and Nutrition Research Project in rural Gaibandha District, northern Bangladesh. JiVitA has conducted multiple food and micronutrient supplement trials in pregnant women and children for over two decades (Klemm et al., 2008; West Jr. et al., 2014, 2011). The JiVitA study area has similar sociodemographic characteristics to rural Bangladesh overall (Labrique et al., 2011). The PAIR Study was designed to assess whether arsenic exposure and micronutrient deficiencies altered maternal or newborn immunity and acute morbidity following maternal seasonal influenza vaccination during pregnancy. Study enrollment has been described in detail elsewhere (Avolio et al., 2023). Briefly, from July 2018 to March 2019, we conducted monthly home visits to married women of reproductive age (13–45 years) in the study area. If a woman reported her last menstrual period was >30 days from the date of the visit, she was offered a urine pregnancy test. A pregnant woman was eligible for the PAIR Study if she was 13–16 weeks of gestational age, had no pre-existing immunodeficiency or chronic infection, had no previous or current use of immune-altering drugs or therapies (*e.g.*, steroids), and had not yet received an influenza vaccine for the 2018–19 influenza season. From October 2018 to March 2019, we enrolled 784 pregnant women. Because of the logistical challenges of conducting visits in a large rural area of Bangladesh, a small number of visits occurred earlier or later than scheduled. Thus, we enrolled 8 (1.0%) women in gestational weeks 11–12, 775 (98.9%) women in weeks 13–16, and 1 (0.1%) woman in week 17. This paper reports a cross-sectional analysis of data collected at enrollment with 765 (97.6%) complete cases. Excluded participants were missing one or more of the following variables: subscapular skinfold thickness (n = 6 [0.77%]), mid-upper arm circumference (n = 1 [0.13%]), urinary arsenic (n = 1 [0.13%]), drinking water arsenic (n = 4 [0.51%]), maternal age (n = 2 [0.26%]), plasma folate (n = 4 [0.51%]), plasma vitamin B12 (n = 1 [0.13%]), and plasma homocysteine (n = 2 [0.26%]). The PAIR Study was approved by the institutional review boards of the Johns Hopkins Bloomberg School of Public Health in Baltimore, Maryland, United States and the Institute for Epidemiology, Disease Control, and Research in Dhaka, Bangladesh. All participants gave informed consent prior to enrollment.

### 2.2 Anthropometry

We assessed anthropometry at enrollment using BMI, subscapular and triceps skinfold thicknesses, and mid-upper arm circumference (MUAC), fat area (MUAFA), and muscle area (MUAMA). BMI (kg/m^2^) was calculated as weight divided by height squared. Weight in light clothing and without shoes was measured to the nearest 100 g using a digital scale (Taylor, Oak Brook, IL USA). Height without shoes was measured to the nearest 0.1 cm using a Cromwell Harpenden Pocket Stadiometer with Spirit Level manufactured locally by JiVitA. Skinfold thickness was measured to the nearest 0.2 mm using Holtain calipers (Crosswell, United Kingdom). MUAC was measured to the nearest 0.1 cm using a non-stretch insertion-type measuring tape manufactured locally by JiVitA. MUAFA (cm^2^) and MUAMA (cm^2^) were derived from triceps skinfold thickness and MUAC using standard formulae:

$$MUAFA=\left(\left(Triceps\;Skinfold\;x\;MUAC\right)\;/\;2\right)\;–\;(\left(\pi \;x\;\left(Triceps\;Skinfol{d)}^{2}\right)\;/\;4\right)$$

$$MUAMA=(\left(MUAC\;–\;{\left(\pi \;x\;Triceps\;Skinfold\right))}^{2}\;/\;4\pi \right)\;–\;6.5$$

(Gibson, 2005). All measurements (except weight, which was measured only once) were taken in triplicate at enrollment, and median values were used for this analysis.

### 2.3 Arsenic methylation

We assessed arsenic methylation at enrollment using urinary arsenic species. Specimen collection, handling, and analysis have been described in detail elsewhere (Avolio et al., 2023). Briefly, spot urine specimens were collected at enrollment and transported on ice packs to a JiVitA field laboratory, where the specimens were aliquoted and stored at −20 °C on the day of collection. Urinary arsenic was speciated (iAs, MMA, and DMA) at the Institute of Chemistry – Analytical Chemistry at the University of Graz in Graz, Austria using high performance liquid chromatography (HPLC) with inductively coupled plasma tandem mass spectrometry (ICP-MS/MS) (Scheer et al., 2012). iAs was the sum of arsenite and arsenate. The mean inter- and intra-assay coefficients of variation (CoVs) for urinary iAs, MMA, and DMA were 5.0% and 1.8%, 5.6% and 2.9%, and 5.0% and 1.6%, respectively. All species were greater than or equal to the lower limit of detection (LLOD) of 0.05 μg/L in all participants. Arsenobetaine and other arsenic cations were low (median [interquartile range (IQR)]: 0.79 [0.38, 1.73] μg/L), indicating that urinary arsenic primarily reflects inorganic arsenic exposure and not organic arsenic exposure from seafood consumption (Navas-Acien et al., 2011). Specific gravity was measured by refractometric determination of total solids. iAs, MMA, and DMA were specific gravity-corrected (Boeniger et al., 1993; Levine and Fahy, 1945), divided by their sum (∑uAs), and multiplied by 100 (iAs%, MMA%, and DMA%). The primary methylation index (PMI) and secondary methylation index (SMI) were calculated as MMA/iAs and DMA/MMA, respectively. The interpretation of arsenic methylation measures is complicated: higher MMA%, for example, may reflect more primary methylation of iAs to MMA or less secondary methylation of MMA to DMA. In general, however, lower iAs% and MMA%, and higher DMA% and SMI, indicate greater arsenic methylation efficiency.

### 2.4 Other variables

We measured participants’ household drinking water arsenic concentrations (wAs), as household drinking water was a primary source of arsenic exposure in our sample (Avolio et al., 2023). Drinking water specimens were collected at enrollment and transported on ice packs to the JiVitA field laboratory, where they were aliquoted and stored at −20 °C on the day of collection. Arsenic was measured at the Lamont-Doherty Earth Observatory of Columbia University in Palisades, New York using inductively coupled plasma mass spectrometry (ICP-MS) (Cheng et al., 2004). The mean inter- and intra-assay CoVs were 2.7% and 1.9%, respectively. For wAs < LLOD of 0.02 μg/L (n = 2 [0.26%]), we imputed LLOD/√2 (Hornung and Reed, 1990).

Maternal age was calculated as the difference in years between the date of enrollment and the self-reported date of birth. Gestational age was calculated as the difference in weeks between the date of enrollment and the self-reported date of last menstrual period. Parity (number of live births prior to the current pregnancy) was assessed by questionnaire and categorized as Nulliparous (0), Primiparous (1), and Multiparous (≥2). Education was assessed by questionnaire and categorized as None, Class 1–9 (some but did not complete secondary education), and Class ≥10 (completed secondary education). Living standards index, a measure of socioeconomic status derived by principal components analysis of household assets and home construction materials, was calculated as described elsewhere (Gunnsteinsson et al., 2010).

We assessed one-carbon metabolism micronutrient status at enrollment using plasma biomarkers. Venous blood specimens were collected at enrollment in tubes containing the anticoagulant sodium heparin and transported on ice packs to the JiVitA field laboratory. Blood was centrifuged to isolate plasma, which was aliquoted and stored at −80 °C on the day of collection. Plasma samples were not thawed prior to micronutrient analyses. Plasma folate, vitamin B12, and homocysteine were measured at the Human Nutrition Laboratory of the Johns Hopkins University in Baltimore, Maryland using chemiluminescent immunoassays (Immulite, 2000; Siemens Diagnostics, Malvern, PA USA). The mean inter- and intra-assay CoVs for plasma folate, vitamin B12, and homocysteine were 10.3% and 4.2%, 7.7% and 3.3%, and 11.1% and 4.7%, respectively. In general, higher plasma folate and vitamin B12 concentrations indicate better micronutrient status. Folate and vitamin B12 deficiencies were defined as <6.8 nmol/L and <150 pmol/L, respectively (Schulze et al., 2019). Plasma homocysteine is inversely associated with circulating folate and vitamin B12, as well as choline (not assessed here); higher concentrations result from an inability to demethylate homocysteine and are indicative of compromised metabolic status. Elevated homocysteine was defined as >15 μmol/L (Dib et al., 2022).

### 2.5 Statistical analysis

We calculated summary statistics for arsenic methylation measures and all covariates and estimated pairwise Spearman's correlation coefficients for anthropometric, arsenic methylation, and micronutrient status measures. We then estimated cross-sectional associations between anthropometric and arsenic methylation measures using linear regression models. Arsenic methylation measures were the dependent variables. Each anthropometric measure was scaled by the IQR of its values among study participants so that coefficients refer to expected differences in the mean of the arsenic methylation measure per IQR-unit difference in the anthropometric measure. PMI and SMI were natural log (ln)-transformed to improve normality (Fig. S1) and coefficients from PMI and SMI models were transformed using (*e*^β^ – 1) x 100 to obtain expected *percent* differences in geometric mean PMI or SMI per IQR-unit difference in the anthropometric measure. The functional forms of anthropometric measures (*e.g.*, linear, quadratic) were assessed by inspecting locally estimated scatterplot smoothers (Figs. S2–S7) and comparing Akaike information criteria for models with and without quadratic terms (Table S1). Ultimately, for each combination of anthropometric measure and arsenic methylation measure, we fitted three models. The first model (“Unadjusted”) included just the anthropometric measure (linear). The second model (“Adjusted 1”) also included wAs (ln μg/L; linear and quadratic), maternal age (years; linear), gestational age (11–13, 14, 15, 16–17 weeks; indicators), education (none, Class 1–9, Class ≥10; indicators), and living standards index (unitless; linear). To assess for confounding by one-carbon metabolism micronutrient status, the third model (“Adjusted 2”) further included plasma folate (ln nmol/L; linear), plasma vitamin B12 (ln pmol/L; linear), and plasma homocysteine (ln μmol/L; linear). To better distinguish the potential roles of lean and fat mass in arsenic methylation, we also fitted linear regression models including MUAFA, MUAMA, and Adjusted 2 confounders as independent variables.

As a sensitivity analysis to assess robustness to the type of regression model, we also estimated associations between anthropometric measures and iAs%, MMA%, and DMA% using beta and Dirichlet regression models, which are more appropriate than linear regression when the dependent variable is a continuous proportion (Ferrari and Cribari-Neto, 2004; Hijazi and Jernigan, 2009). Beta regression assumes the dependent variable follows a beta distribution, which is defined on the interval [0, 1] and parameterized by μ (expected value) and φ (precision) (Douma and Weedon, 2019). Dirichlet regression is a multivariate extension of beta regression in which μ is a vector of proportions that sum to one (Douma and Weedon, 2019). Dirichlet models omit one of the response variables (here, iAs%), which is estimated implicitly. iAs%, MMA%, and DMA% were scaled to [0, 1] by dividing by 100, but we have used the same names throughout for clarity. Both μ and φ can be estimated conditional on covariates, and conditioning φ on the denominator of the proportion can improve model fit when the denominator varies among observations (Douma and Weedon, 2019; Smithson and Verkuilen, 2006). Accordingly, φ was modeled conditional on ln ∑uAs. For the beta and Dirichlet models, we used logit link functions, and coefficients refer to expected differences in the log odds of an arsenic methylation proportion per IQR-unit difference in an anthropometric measure. Since differences in the log odds of continuous proportions may be less interpretable than differences in the means, we focused on whether there was consistency in the direction of estimates relative to null values across linear, beta, and Dirichlet models. All statistical analyses were conducted in R (R Project for Statistical Computing, Vienna, Austria). We used the betareg and DirichletReg packages for beta and Dirichlet regression, respectively (Cribari-Neto and Zeileis, 2010; Maier, 2014). All code is available on GitHub (https://www.github.com/tylerjssmith).

## 3 Results

The included participants (n = 765) were women of reproductive age (IQR: 23, 30 years) in the early second trimester (IQR: 13, 15 weeks) who were predominantly primiparous (46.1%) or multiparous (35.8%) prior to the current pregnancy (Table 1). The majority had some primary or secondary education (69.9%) or had completed secondary education (18.3%) (Table 1). Vitamin B12 deficiency (49.0%) was more common than folate deficiency (19.2%) (Table 1). Despite these, elevated homocysteine was rare (1.4%) (Table 1). Median (IQR) BMI was 21.5 (19.4, 23.8) kg/m^2^. Median (IQR) subscapular and triceps skinfold thicknesses were 17.8 (13.2, 24.2) mm and 14.2 (10.2, 18.6) mm, respectively. Median (IQR) MUAC, MUAFA, and MUAMA were 25.9 (23.8, 28.0) cm, 15.3 (10.5, 20.3) cm^2^, and 29.9 (25.6, 34.2) cm^2^, respectively (Table 2). Both drinking water arsenic (median [IQR]: 5.1 [0.5, 25.1] μg/L) and ∑uAs (median [IQR]: 33.3 [19.5, 56.9]) indicated low-moderate arsenic exposure among study participants (Table 1). Median (IQR) iAs%, MMA%, and DMA% were 12.0 (9.3, 15.2)%, 6.6 (5.3, 8.3)%, and 81.0 (77.1, 84.6)%, respectively (Table 2). Median (IQR) PMI and SMI were 0.6 (0.4, 0.7) and 12.2 (9.3, 15.7), respectively (Table 2).

**Table 1:** Characteristics of Pregnant Women (n = 765) at Enrollment in the PAIR Study, Gaibandha District, Bangladesh, 2018–2019 Summary statistics are median (IQR) for continuous variables and n (%) for discrete variables. Abbreviations: BMI, body mass index, IQR, interquartile range; ∑uAs, sum of urinary inorganic and methylated arsenic species; wAs, drinking water arsenic.

		BMI Category (kg/m2)		
	Overall	<18.5	≥18.5 to <25	≥25
Overall		117 (15.3)	522 (68.2)	126 (16.5)
Age (years)	26 (23, 30)	25 (21, 29)	26 (23, 30)	28 (24.2, 30)
Gestational Age (weeks)	14 (13, 15)	14 (13, 14)	14 (13, 15)	14 (13, 15)
Parity				
Nulliparous	138 (18.0)	26 (22.2)	94 (18.0)	18 (14.3)
Primiparous	353 (46.1)	47 (40.2)	250 (47.9)	56 (44.4)
Multiparous	274 (35.8)	44 (37.6)	178 (34.1)	52 (41.3)
Education				
None	90 (11.8)	18 (15.4)	61 (11.7)	11 (8.7)
Class 1–9	535 (69.9)	88 (75.2)	369 (70.7)	78 (61.9)
Class ≥10	140 (18.3)	11 (9.4)	92 (17.6)	37 (29.4)
wAs (μg/L)	5.1 (0.5, 25.1)	4.4 (0.7, 21.8)	5.2 (0.5, 24.7)	5.9 (0.4, 34)
∑uAs (μg/L)	33.3 (19.5, 56.9)	35.3 (22.1, 54.6)	32.3 (19.3, 56.1)	33.7 (19.4, 60.6)
Plasma Folate (nmol/L)	10.1 (7.4, 13.8)	8.8 (6.6, 12.2)	10.1 (7.6, 13.8)	11.2 (7.8, 14.9)
Folate Status				
Sufficient (≥6.8 nmol/L)	618 (80.8)	84 (71.8)	433 (83.0)	101 (80.2)
Deficient (<6.8 nmol/L)	147 (19.2)	33 (28.2)	89 (17.0)	25 (19.8)
Plasma Vitamin B12 (pmol/L)	151.2 (111.4, 202.9)	163.8 (129.1, 211.0)	147.8 (109.0, 199.0)	150.9 (114.4, 205.7)
Vitamin B12 Status				
Sufficient (≥150 pmol/L)	390 (51.0)	70 (59.8)	256 (49.0)	64 (50.8)
Deficient (<150 pmol/L)	375 (49.0)	47 (40.2)	266 (51.0)	62 (49.2)
Plasma Homocysteine (μmol/L)	5.7 (4.6, 7.1)	5.8 (4.9, 7.2)	5.7 (4.6, 7.0)	5.7 (4.4, 7.0)
Homocysteine Status				
Normal (≤15 μmol/L)	754 (98.6)	113 (96.6)	516 (98.9)	125 (99.2)
Elevated (>15 μmol/L)	11 (1.4)	4 (3.4)	6 (1.1)	1 (0.8)

**Table 2:** Anthropometric and Arsenic Methylation Measures among Pregnant Women (n = 765) at Enrollment in the PAIR Study, Gaibandha District, Bangladesh, 2018–2019 Median (IQR) gestational age at enrollment was 14 (13, 15) weeks. Abbreviations: DMA%, dimethyl arsenic percentage; iAs%, inorganic arsenic percentage; IQR, interquartile range; MMA%, monomethyl arsenic percentage; PMI, primary methylation index (MMA/iAs); SD, standard deviation; SMI, secondary methylation index (DMA/MMA).

	Mean (SD)	Minimum	Quartile 1	Median	Quartile 3	Maximum
Body Mass Index (kg/m^2^)	21.8 (3.3)	15.2	19.4	21.5	23.8	36.0
Weight (kg)	49.0 (8.3)	31.7	42.8	48.0	54.2	83.9
Height (cm)	149.8 (5.2)	131.4	146.1	149.9	153.2	165.9
Subscapular Skinfold Thickness (mm)	19.0 (7.5)	4.4	13.2	17.8	24.2	40.0
Triceps Skinfold Thickness (mm)	14.8 (5.7)	4.0	10.2	14.2	18.6	36.0
Mid-upper Arm Circumference (cm)	26.0 (3.0)	18.6	23.8	25.9	28.0	38.8
Mid-upper Arm Fat Area (cm^2^)	16.0 (6.8)	3.7	10.5	15.3	20.3	46.6
Mid-upper Arm Muscle Area (cm^2^)	30.2 (6.4)	9.2	25.6	29.9	34.2	71.7
iAs%	12.6 (4.7)	1.1	9.3	12.0	15.2	35.6
MMA%	7.0 (2.4)	1.9	5.3	6.6	8.3	21.8
DMA%	80.4 (6.1)	52.0	77.1	81.0	84.6	96.8
Primary Methylation Index	0.61 (0.24)	0.2	0.4	0.6	0.7	1.9
Secondary Methylation Index	13.1 (5.7)	2.8	9.3	12.2	15.7	50.9

Spearman's correlations between anthropometric and arsenic methylation measures were weak but consistent across anthropometric measures. Anthropometric measures were negatively correlated with iAs% (range of pairwise Spearman correlations: -0.15, −0.08) and MMA% (−0.22, −0.11) and positively correlated with DMA% (0.11, 0.20) and SMI (0.11, 0.23) (Table 3). The correlations with PMI were much weaker (−0.05, −0.02) (Table 3). Spearman's correlations were less consistent across micronutrient status measures. Plasma folate was negatively correlated with iAs% (−0.13) and positively correlated with DMA% (0.12) and PMI (0.11) (Table 3). Plasma vitamin B12 was negatively correlated with iAs% (−0.08) and positively correlated with PMI (0.14) (Table 3). Plasma homocysteine was positively correlated with iAs% (0.10) and negatively correlated with DMA% (−0.11) and SMI (−0.08) (Table 3). Taken together, these correlations suggest that anthropometric measures were more strongly correlated with SMI than PMI, while micronutrient status measures were more strongly correlated with PMI than SMI. As expected, except for MUAMA, anthropometric measures were strongly and positively correlated with each other (range: 0.76, 0.98) (Table 3). MUAMA was strongly correlated with MUAC (0.83) but only moderately correlated with other anthropometric measures (<0.70) (Table 3). Anthropometric measures were mostly uncorrelated with micronutrient status measures. The notable exceptions were the negative correlation between MUAMA and plasma vitamin B12 (−0.10) and the positive correlation between MUAMA and plasma homocysteine (0.08) (Table 3).

**Table 3:** Pairwise Spearman's Correlation Coefficients for Arsenic Methylation, Anthropometric, and Micronutrient Status Measures among Pregnant Women (n = 765) at Enrollment in the PAIR Study, Gaibandha District, Bangladesh, 2018–2019 Median (IQR) gestational age at enrollment was 14 (13, 15) weeks. *, **, and *** indicate p-value <0.05, <0.01, and <0.001, respectively. Abbreviations: BMI, body mass index; B12, plasma vitamin B12; DMA%, dimethyl arsenic percentage; Folate, plasma folate; Hcy, plasma homocysteine; iAs%, inorganic arsenic percentage; IQR, interquartile range; MMA%, monomethyl arsenic percentage; MUAC, mid-upper arm circumference; MUAFA, mid-upper arm fat area; MUAMA, mid-upper arm muscle area; PMI, primary methylation index (MMA/iAs); SMI, secondary methylation index (DMA/MMA); Subscapular, subscapular skinfold thickness; Triceps, triceps skinfold thickness.

	iAs%	MMA%	DMA%	PMI	SMI	BMI	Subscapular	Triceps	MUAC	MUAFA	MUAMA	Folate	B12	Hcy
iAs%	1.00													
MMA%	*** 0.42	1.00												
DMA%	*** −0.93	*** −0.71	1.00											
PMI	*** −0.62	*** 0.38	*** 0.31	1.00										
SMI	*** −0.53	*** −0.99	*** 0.79	*** −0.27	1.00									
BMI	*** −0.15	*** −0.22	*** 0.20	−0.05	*** 0.23	1.00								
Subscapular	*** −0.12	*** −0.18	*** 0.17	−0.03	*** 0.18	*** 0.78	1.00							
Triceps	*** −0.14	*** −0.19	*** 0.19	−0.02	*** 0.19	*** 0.82	*** 0.82	1.00						
MUAC	*** −0.12	*** −0.16	*** 0.17	−0.03	*** 0.17	*** 0.88	*** 0.76	*** 0.82	1.00					
MUAFA	*** −0.14	*** −0.18	*** 0.19	−0.02	*** 0.19	*** 0.87	*** 0.84	*** 0.98	*** 0.91	1.00				
MUAMA	* −0.08	** −0.11	** 0.11	−0.02	** 0.11	*** 0.68	*** 0.46	*** 0.39	*** 0.83	*** 0.55	1.00			
Folate	*** −0.13	−0.03	** 0.12	** 0.11	0.05	0.04	0.03	0.07	0.03	0.06	−0.02	1.00		
B12	* −0.08	0.07	0.03	*** 0.14	−0.06	−0.07	−0.02	−0.02	−0.07	−0.03	** −0.10	0.04	1.00	
Hcy	** 0.10	0.06	** −0.11	−0.06	* −0.08	−0.02	−0.06	−0.05	0.02	−0.03	* 0.08	*** −0.39	*** −0.17	1.00

**Fig. 1:**
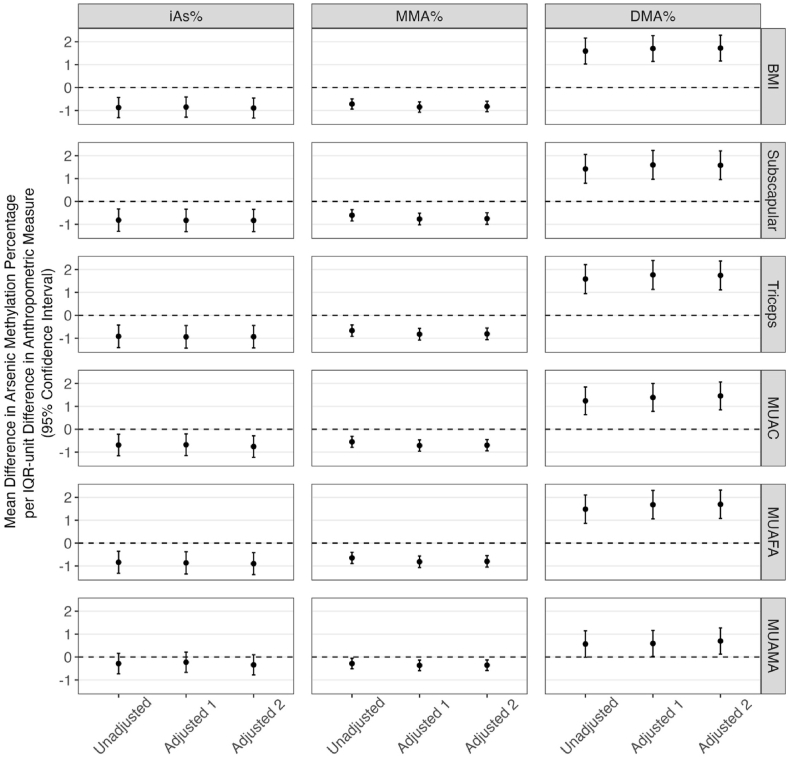
Linear Regression Estimates of Mean Differences in Arsenic Methylation Percentages per IQR-unit Differences in Anthropometric Measures among Pregnant Women (n = 765) at Enrollment in the PAIR Study, Gaibandha District, Bangladesh, 2018-2019 Median (IQR) gestational age at enrollment was 14 (13, 15) weeks. Unadjusted models contained just the anthropometric measure. Adjusted 1 models also included drinking water arsenic, age, gestational age, education, and living standards index. Adjusted 2 models further included plasma folate, plasma vitamin B12, and plasma homocysteine. Abbreviations: BMI, body mass index; DMA%, dimethyl arsenic percentage; iAs%, inorganic arsenic percentage; IQR, interquartile range; MMA%, monomethyl arsenic percentage; MUAC, mid-upper arm circumference; MUAFA, mid-upper arm fat area; MUAMA, mid-upper arm muscle area; Subscapular, subscapular skinfold thickness; Triceps, triceps skinfold thickness.

**Fig. 2:**
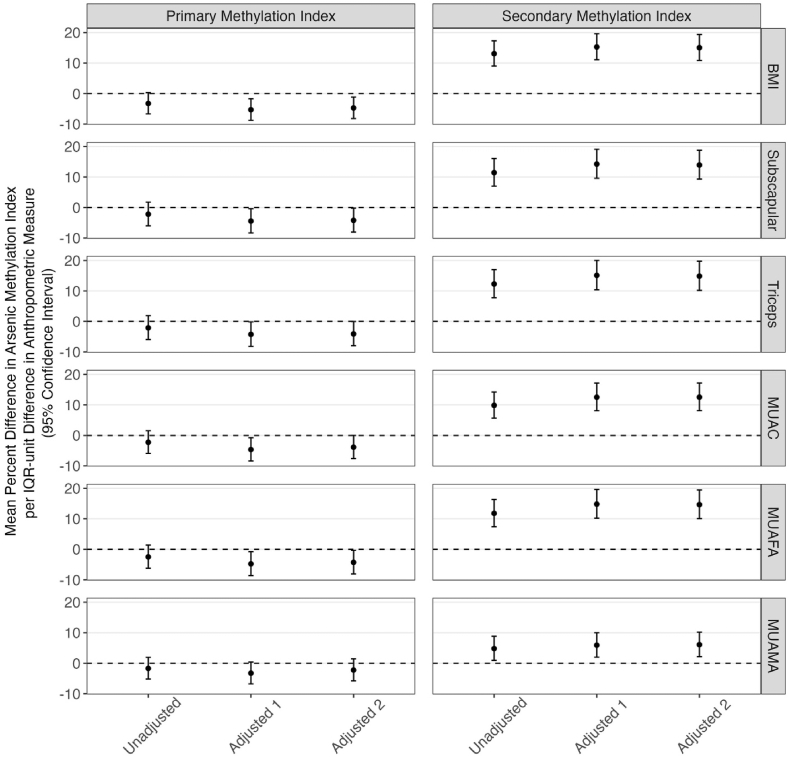
Linear Regression Estimates of Mean Percent Differences in Arsenic Methylation Indices per IQR-unit Differences in Anthropometric Measures among Pregnant Women (n = 765) at Enrollment in the PAIR Study, Gaibandha District, Bangladesh, 2018-2019 Median (IQR) gestational age at enrollment was 14 (13, 15) weeks. Unadjusted models contained just the anthropometric measure. Adjusted 1 models also included drinking water arsenic, age, gestational age, education, and living standards index. Adjusted 2 models further included plasma folate, plasma vitamin B12, and plasma homocysteine. BMI, body mass index; IQR, interquartile range; MUAC, mid-upper arm circumference; MUAFA, mid-upper arm fat area; MUAMA, mid-upper arm muscle area; Subscapular, subscapular skinfold thickness; Triceps, triceps skinfold thickness.

In linear regression models, across adjustment sets, BMI, subscapular skinfold thickness, triceps skinfold thickness, and MUAC were negatively associated with iAs%, MMA%, and PMI and positively associated with DMA% and SMI. Moreover, all associations persisted or strengthened after adjustment for potential confounders, including the plasma biomarkers of micronutrient status. For example, in Unadjusted models, mean differences (95% confidence intervals [CI]) in iAs% per IQR-unit difference in BMI, subscapular skinfold thickness, triceps skinfold thickness, and MUAC were −0.87 (−1.32, −0.43), −0.82 (−1.30, −0.33), −0.92 (−1.41, −0.43), and −0.69 (−1.16, −0.22) pp, respectively (Fig. 1, Table S2). In Adjusted 2 models, mean differences (95% CI) in iAs% were −0.89 (−1.33, −0.46), −0.83 (−1.32, −0.34), −0.93 (−1.42, −0.45), and −0.76 (−1.23, −0.28) pp, respectively (Fig. 1, Table S2). Conversely, in Unadjusted models, mean differences (95% CI) in DMA% per IQR-unit difference in BMI, subscapular skinfold thickness, triceps skinfold thickness, and MUAC were 1.59 (1.03, 2.16), 1.42 (0.79, 2.05), 1.59 (0.95, 2.22), and 1.24 (0.64, 1.84) pp, respectively (Fig. 1, Table S2). In Adjusted 2 models, mean differences in DMA% were 1.72 (1.16, 2.28), 1.58 (0.95, 2.21), 1.74 (1.11, 2.37), and 1.45 (0.85, 2.06) pp, respectively (Fig. 1, Table S2). While associations with PMI were negative, they were not statistically significant in some models. In the Adjusted 2 models, for example, mean percent differences in PMI per IQR-unit difference in BMI, subscapular skinfold thickness, triceps skinfold thickness, and MUAC were −4.71 (−8.15, −1.14)%, −4.21 (−8.04, −0.22)%, −4.09 (−7.95, −0.06)%, and −3.87 (−7.59, 0.01)%, respectively (Fig. 2, Table S2).

In linear regression models that included MUAFA or MUAMA but not both, each measure was negatively associated with iAs%, MMA%, and PMI and positively associated with DMA% and SMI, although associations between MUAMA and arsenic methylation measures were consistently weaker and sometimes not statistically significant. For example, in MUAFA-only models, the Unadjusted and Adjusted 2 mean differences in iAs% per IQR-unit difference in MUAFA were −0.84 (−1.32, −0.36) and −0.90 (−1.38, −0.42), respectively (Fig. 1, Table S2). The Unadjusted and Adjusted 2 mean differences in DMA% per IQR-unit difference in MUAFA were 1.48 (0.86, 2.10) and 1.70 (1.08, 2.31), respectively (Fig. 1, Table S2). In MUAMA-only models, the Unadjusted and Adjusted 2 mean differences in iAs% per IQR-unit difference in MUAMA were −0.28 (−0.73, 0.16) and −0.34 (−0.78, 0.10), respectively (Fig. 1, Table S2). The Unadjusted and Adjusted 2 mean differences in DMA% per IQR-unit difference in MUAMA were 0.57 (−0.01, 1.15) and 0.70 (0.13, 1.27), respectively (Fig. 1, Table S2). In models that included both MUAFA and MUAMA, however, associations between MUAFA and arsenic methylation measures persisted or strengthened while associations between MUAMA and arsenic methylation measures attenuated. In these methods, mean differences in iAs% per IQR-unit differences in MUAFA and MUAMA were −0.95 (−1.51, −0.39) and 0.10 (−0.41, 0.60), respectively (Table S3). The mean differences in DMA% per IQR-unit differences in MUAFA and MUAMA were 1.76 (1.04, 2.48) and −0.11 (−0.77, 0.54), respectively (Table S3).

In the sensitivity analyses, the same patterns (negative associations with iAs% and MMA%, positive associations with DMA%) were observed in beta regression models (Table S4, Fig. S8). In Dirichlet regression models, associations with MMA% were negative but not statistically significant while associations with DMA% were positive and statistically significant (Table S5, Fig. S9). Like in linear models, in beta and Dirichlet models that included both MUAFA and MUAMA, associations between MUAFA and arsenic methylation measures persisted or strengthened while associations between MUAMA and arsenic methylation measures attenuated (Table S6, Table S7).

## 4 Discussion

The results of our study partly confirm our hypothesis. First, we observed consistent cross-sectional associations between anthropometric and arsenic methylation measures among pregnant women in rural northern Bangladesh. In our study, negative associations with iAs% and MMA% and positive associations with DMA% and SMI suggest that anthropometric measures were positively associated with arsenic methylation efficiency in the early second trimester. A notable inconsistency was negative associations with PMI, which measures primary methylation of iAs to MMA. This may suggest that anthropometric measures are more strongly associated with secondary methylation of MMA to DMA than with primary methylation. Second, and contrary to our hypothesis, none of the associations were attenuated by adjustment for biomarkers of micronutrient status; in most cases, fully adjusted estimates were stronger than unadjusted estimates. This suggests that positive associations between anthropometric measures and arsenic methylation were not attributable to confounding by micronutrient status. Indeed, anthropometric and micronutrient status measures were mostly uncorrelated. Moreover, while anthropometric measures were correlated with SMI more than PMI, micronutrient status measures were correlated with PMI more than SMI. This suggests one-carbon metabolism micronutrient status may be more related to primary methylation (iAs → MMA) and anthropometric measures may be more related to secondary methylation (MMA → DMA). Finally, in models that included both MUAFA and MUAMA, persistence of MUAFA associations and attenuation of MUAMA associations suggest that adiposity, more than lean mass, may explain associations between anthropometric measures and arsenic methylation.

These results are consistent with previous reports of positive associations between BMI and arsenic methylation in non-pregnant adults (Abuawad et al., 2021; Bommarito et al., 2019; Gamboa-Loira et al., 2021; Gomez-Rubio et al., 2011; Gribble et al., 2013) and may clarify the relationship between BMI and arsenic methylation in pregnancy. To our knowledge, three previous studies have estimated associations between BMI and arsenic methylation in pregnant women. In Spain, Soler-Blasco et al. measured BMI prior to pregnancy and arsenic methylation in the late first or early second trimester (n = 1017; mean [SD] gestational age: 13.0 [1.2] weeks) and reported negative associations between BMI and iAs% and MMA% and positive associations between BMI and DMA% (Soler-Blasco et al., 2021), which is consistent with our study in northern Bangladesh. By contrast, in southeastern Bangladesh, Li et al. measured both BMI and arsenic methylation in the first trimester (n = 442; mean [10th, 90th percentile] gestational age: 8.0 [5.9, 11.4] weeks) and did not find statistically significant (*p* < 0.05) associations between BMI and iAs%, MMA%, DMA%, PMI, or SMI (Li et al., 2008). The authors did not report their point estimates, however, and the lower sample size may explain the lack of statistical significance. In central Bangladesh, Gao et al. measured BMI and arsenic methylation in the late first or early second trimester (n = 1606; mean [SD] gestational age: 11.0 [3.0] weeks) and found a negative association with MMA% but no associations with iAs% or DMA% (Gao et al., 2019). In the same study, early-pregnancy BMI also was not associated with any of the arsenic methylation percentages in the third trimester (n = 1445; mean [SD] gestational age: 28.9 [1.9] weeks) (Gao et al., 2019).

Several factors may explain the different results across these studies. First, the only study to report no association between BMI and any measure of arsenic methylation (Li et al., 2008) had a notably smaller sample size than the others. Second, Gomez-Rubio et al. have proposed that differences in BMI distributions may explain differences in associations between BMI and arsenic methylation across studies of non-pregnant adults, with the associations we observed more likely in samples with higher prevalence of overweight and obesity (higher BMI) (Gomez-Rubio et al., 2011). A comparison of studies in pregnant women is complicated because BMI was measured at different times. Differences between first- and second-trimester BMI, for example, may represent varying contributions from materno-placento-fetal growth as well as early plasma volume expansion, and thus reflect variable increases in lean and fat mass. Moreover, even in non-pregnant adults, the relationship between adiposity and BMI is known to vary appreciably among populations (Shaikh et al., 2013, 2016). Still, just 5.4% of participants in Li et al. had BMI >25 kg/m^2^ (Li et al., 2008), compared to 16.6% in our study and 23% in Soler-Blasco et al. (2021). Gao et al. did not report BMI categories, but the mean (SD) BMI was lower in their sample (21.0 [3.0] kg/m^2^) than in ours (21.8 [3.3] kg/m^2^). Thus, our results may be more applicable to populations with high prevalence of overweight and obesity. Third, associations may differ by the timing of BMI or arsenic methylation measurements. Across three previous studies and ours, BMI was measured between pre-pregnancy and the second trimester and arsenic methylation was measured between the first and third trimesters. A pattern based on the time of BMI measurement is not apparent, as the most consistent associations were reported by Soler-Blasco et al. (pre-pregnancy BMI) and us (early second-trimester BMI). Less consistent associations were reported by Li et al. and Gao et al. (first-trimester or first- and second-trimester BMI). By contrast, arsenic methylation was measured later in our study (mean: 14 gestational weeks) and by Soler-Blasco et al. (mean: 13 weeks) than in Li et al. (mean: 8 weeks) and Gao et al. (mean: 11 weeks). How associations between anthropometric and arsenic methylation measures may vary over pregnancy deserves further study. Notably, Gao et al. reported no association between early-pregnancy BMI and late-pregnancy arsenic methylation; thus, associations may vary non-monotonically with gestational age.

Because of our cross-sectional study design, we cannot establish the direction of a causal relationship, if any, between anthropometric measures and arsenic methylation. However, there are plausible biological mechanisms by which adiposity, in particular, may promote arsenic methylation. Arsenic methylation depends on several interconnected pathways related to one-carbon metabolism (for an accessible review with an emphasis on pregnancy, see (Vahter, 2009)). Briefly, dietary protein is a source of the amino acid methionine, which is activated to produce the methyl donor S-adenosylmethionine (SAM). Methyl groups are transferred from SAM to iAs or MMA in a reaction catalyzed by the enzyme AS3MT. This reaction yields MMA (if the substrate was iAs) or DMA (if the substrate was MMA) as well as S-adenosyl-L-homocysteine (SAH). SAH is converted to homocysteine. Since homocysteine inhibits arsenic methylation, it must be efficiently removed by remethylation back to methionine, which may be activated again to produce SAM. SAH remethylation may be accomplished by a folate and vitamin B12-dependent pathway or a betaine-dependent pathway (Vahter, 2009) with the betaine-dependent pathway becoming increasingly important over time in pregnancy (Velzing-Aarts et al., 2005). Betaine is derived from choline, and choline synthesis is upregulated by estrogen (Resseguie et al., 2007). Since adipose tissue is estrogenic (Kershaw and Flier, 2004), Abuawad et al. have proposed the causal pathway adiposity → estrogen → choline → arsenic methylation (Abuawad et al., 2021; Bustaffa et al., 2020). They cautioned, however, that choline may promote adiposity (Zeisel, 2013), and temporality among adiposity, choline, and arsenic methylation remains unclear (Abuawad et al., 2021). Additional longitudinal studies incorporating specific measures of adiposity are needed to establish temporality and assess mechanisms.

Strengths of this study include taking measurements at approximately the same gestational age, using multiple anthropometric measures and micronutrient status biomarkers, and demonstrating consistency across types of regression models. First, as noted above, associations between anthropometric measures and arsenic methylation may depend on the time of measurement. In our study, measurements were taken within a narrow gestational age range (median [IQR]: 14 [13, 15] weeks), reducing differences among participants with respect to potential confounders. Second, we found that six anthropometric measures were consistently associated with arsenic methylation. This overcomes some of the ambiguity of BMI and increases our confidence that associations reflect a relationship between anthropometric measures, including adiposity, and arsenic methylation. Third, previous studies of BMI and arsenic methylation in pregnancy have adjusted for estimates of micronutrient intake derived from food frequency questionnaires. Biomarkers of micronutrient status, however, also reflect changing physiological requirements in pregnancy (Bae et al., 2015) and may better measure the availability of one-carbon metabolism micronutrients. We found that associations were not attenuated by adjustment for these biomarkers. Fourth, when response variables are continuous proportions, the assumptions of linear regression models may be violated (Douma and Weedon, 2019). Beta and Dirichlet models are more appropriate in these contexts, but their estimands (expected differences in log odds) may be less interpretable. We found consistent results across linear, beta, and Dirichlet models, providing interpretable estimates while showing our conclusions were robust to the type of regression model. There were limitations as well. First, we used a cross-sectional study design without repeated measures of arsenic methylation over time. Arsenic methylation efficiency is known to increase with gestational age (Concha et al., 1998; Hopenhayn et al., 2003; Vahter, 2009). This increased efficiency may protect the developing fetus from arsenic toxicity in late pregnancy (Vahter, 2009). Gardner et al. have reported that BMI measured in the first trimester was not associated with changes in arsenic methylation percentages between the first and third trimesters (Gardner et al., 2011). However, additional longitudinal studies assessing the determinants of change in arsenic methylation during pregnancy are needed. These determinants could include pre- or early-pregnancy anthropometric measures, as in many studies conducted to date, as well as pregnancy-related changes like gestational weight gain. Second, like many studies (*e.g.*, Bommarito et al., 2019; Li et al., 2008; Soler-Blasco et al., 2021), we used urinary arsenic species to assess arsenic methylation. After absorption, arsenic methylation occurs primarily in the liver. While urinary and blood arsenic concentrations have been strongly correlated in previous studies in Bangladesh (Hall et al., 2006), the relative concentrations of urinary arsenic species may not perfectly measure the relative concentrations of these compounds in blood or tissues. The correlation of the relative concentrations in urine and blood could vary among participants by several factors, including variability in renal function (Peters et al., 2015).

Understanding the public health implications of our results and any causal relationship between adiposity and arsenic methylation in pregnancy will require further research on arsenic methylation as a modifier of associations between arsenic exposure and adverse maternal and child health outcomes. We noted in the introduction that, in non-pregnant adults, the role of arsenic methylation appears to vary across cancer, cardiovascular disease, and diabetes mellitus (Kuo et al., 2017). A small number of studies have found evidence that higher arsenic methylation efficiency in pregnancy may protect against certain adverse outcomes (Laine et al., 2015; Signes-Pastor et al., 2021, 2022), but research on these and additional outcomes in pregnant women and children is needed. Potential determinants of arsenic methylation in pregnancy (*e.g.*, maternal adiposity, micronutrient status) may have direct effects on these outcomes, too. Thus, in studies estimating associations between arsenic methylation and maternal and child health outcomes, maternal adiposity may be an important confounder. Maternal BMI may be the only measure of maternal adiposity available. In our study, BMI was highly correlated with most other anthropometric measures and similarly associated with arsenic methylation measures. However, the relationship between BMI and adiposity varies across populations (Shaikh et al., 2016) and between early- and late-pregnancy, and the use of BMI to control for maternal adiposity should be carefully evaluated. Finally, future studies should evaluate the possibility raised by our results that one-carbon metabolism micronutrient status is related to primary methylation of iAs to MMA while anthropometric measures, especially adiposity measures, are related to secondary methylation of MMA to DMA.

## 5 Conclusions

Anthropometric measures were positively associated with arsenic methylation efficiency among pregnant women in the early second trimester in rural northern Bangladesh. Associations were consistent across multiple anthropometric measures and independent of one-carbon metabolism micronutrient status assessed by plasma biomarkers. Our results suggest that adiposity, more than lean mass, may explain associations between anthropometric measures and arsenic methylation. Arsenic methylation is an important determinant of arsenic toxicity in pregnancy and early life, although its role may vary by outcome. Longitudinal studies are needed to assess the determinants of change in arsenic methylation during pregnancy; to establish the direction of a causal relationship, if any, between adiposity and arsenic methylation; to assess plausible biological mechanisms (*e.g.*, adiposity → estrogen → choline → arsenic methylation); and to understand the implications of these results for maternal and child health.

## Credit author statement

TJSS: Writing – Original Draft, Writing – Review & Editing, Conceptualization, Data Curation, Formal Analysis, Software, Visualization; ANA: Writing – Review & Editing, Methodology, Supervision; SB: Writing – Review & Editing, Investigation, Methodology; CK: Investigation; KK: Investigation; LNA: Writing – Review & Editing, Data Curation, Methodology; NP: Writing – Review & Editing, Methodology, Data Curation, Project Administration; PRR: Methodology, Project Administration; RCF: Writing – Review & Editing; WG: Writing – Review & Editing, Investigation, Methodology; AvG: Writing – Review & Editing, Investigation, Methodology; JPB: Writing – Review & Editing, Supervision; MHR: Investigation, Methodology, Supervision; HA: Investigation, Methodology, Project Administration, Supervision; RH: Data Curation, Methodology, Supervision; SS: Methodology, Project Administration, Supervision; TS: Writing – Review & Editing; KS: Writing – Review & Editing, Methodology, Supervision; KPW: Writing – Review & Editing, Funding Acquisition, Methodology, Supervision ABL: Funding Acquisition, Project Administration, Supervision; CDH: Funding Acquisition, Investigation, Methodology, Project Administration, Resources, Supervision.

## Funding

The PAIR Study was supported by the 10.13039/100000066National Institute of Environmental Health Sciences (NIEHS; R01ES026973) and by an unrestricted grant from 10.13039/100014588Sanofi Pasteur, Lyon, France. The PAIR Study also benefited from JiVitA infrastructure and staff supported by the 10.13039/501100008391UBS Optimus Foundation and the 10.13039/100000865Bill & Melinda Gates Foundation (OPP-1141435). TJSS was supported by 10.13039/100000066NIEHS (T32ES007141). ANA was supported by 10.13039/100000066NIEHS (P42ES033719, P30ES009089). JPB was supported by 10.13039/100000066NIEHS (U01ES029531).

## Ethical review

The PAIR Study was approved by the institutional review boards of the Johns Hopkins Bloomberg School of Public Health in Baltimore, Maryland (00008247) and the Institute for Epidemiology, Disease Control, and Research in Dhaka, Bangladesh (IEDCR/IRB/2017/07). All participants gave informed consent prior to enrollment.

## Declaration of competing interest

The authors declare that they have no known competing financial interests or personal relationships that could have appeared to influence the work reported in this paper.

## Data Availability

The data that has been used is confidential.
