# Supplementary material for: Anthropometric measures and arsenic methylation among pregnant women in rural northern Bangladesh
Source: Environ Res. 2023 Oct 1;234:116453. doi: 10.1016/j.envres.2023.116453 (PMC10518461; doi:10.1016/j.envres.2023.116453)

**Supplemental tables and figures for Smith et al. (2023). Anthropometric measures and arsenic methylation among pregnant women in rural northern Bangladesh. *Environmental Research*.**

**Table S1. Akaike Information Criteria (AIC) for Linear Regression Models of Mean Differences in Arsenic Methylation Percentages per IQR-unit Differences in Anthropometric Measures among Pregnant Women (n=765) at Enrollment in the PAIR Study, Gaibandha District, Bangladesh, 2018-2019**

Table compares AIC for models where anthropometric measure was entered as linear term and models where anthropometric measure was entered with linear and quadratic terms. All models included the anthropometric measure, age (linear), gestational age at enrollment (indicators), education (indicators), living standards index (linear), plasma folate (linear), plasma vitamin B12 (linear), and plasma homocysteine (linear). Abbreviations: AIC, Akaike information criterion; iAs%, inorganic arsenic percentage; MMA%, monomethyl arsenic percentage; DMA%, dimethyl arsenic percentage; PMI, primary methylation index (MMA/iAs); SMI, secondary methylation index (DMA/MMA); BMI, body mass index; Subscapular, subscapular skinfold thickness; Triceps, triceps skinfold thickness; MUAC, mid-upper arm circumference; MUAFA, mid-upper arm fat area; MUAMA, mid-upper arm muscle area

|  |  | AIC | |  |
| --- | --- | --- | --- | --- |
| Anthropometric Measure | Arsenic Methylation Measure | Linear | Quadratic | Lower AIC |
| BMI | iAs% | 4510.8 | 4512.8 | Linear |
| BMI | MMA% | 3481.1 | 3483.0 | Linear |
| BMI | DMA% | 4903.2 | 4905.2 | Linear |
| BMI | ln PMI | 667.8 | 669.2 | Linear |
| BMI | ln SMI | 716.9 | 718.2 | Linear |
| Subscapular Skinfold | iAs% | 4515.3 | 4515.4 | Linear |
| Subscapular Skinfold | MMA% | 3497.8 | 3499.8 | Linear |
| Subscapular Skinfold | DMA% | 4913.8 | 4914.7 | Linear |
| Subscapular Skinfold | ln PMI | 670.2 | 670.4 | Linear |
| Subscapular Skinfold | ln SMI | 732.7 | 734.6 | Linear |
| Triceps Skinfold | iAs% | 4511.9 | 4513.7 | Linear |
| Triceps Skinfold | MMA% | 3491.7 | 3493.6 | Linear |
| Triceps Skinfold | DMA% | 4907.9 | 4909.7 | Linear |
| Triceps Skinfold | ln PMI | 670.8 | 672.3 | Linear |
| Triceps Skinfold | ln SMI | 727.1 | 729.1 | Linear |
| MUAC | iAs% | 4516.3 | 4518.2 | Linear |
| MUAC | MMA% | 3500.1 | 3501.8 | Linear |
| MUAC | DMA% | 4915.7 | 4917.5 | Linear |
| MUAC | ln PMI | 670.8 | 672.2 | Linear |
| MUAC | ln SMI | 737.0 | 737.8 | Linear |
| MUAFA | iAs% | 4512.9 | 4514.5 | Linear |
| MUAFA | MMA% | 3492.0 | 3493.9 | Linear |
| MUAFA | DMA% | 4909.1 | 4910.8 | Linear |
| MUAFA | ln PMI | 670.1 | 671.3 | Linear |
| MUAFA | ln SMI | 727.4 | 729.4 | Linear |
| MUAMA | iAs% | 4522.1 | 4523.2 | Linear |
| MUAMA | MMA% | 3519.6 | 3520.5 | Linear |
| MUAMA | DMA% | 4928.8 | 4929.5 | Linear |
| MUAMA | ln PMI | 673.4 | 675.4 | Linear |
| MUAMA | ln SMI | 757.7 | 757.6 | Quadratic |

**Table S2. Linear Regression Estimates of Mean Differences in Arsenic Methylation Measures per IQR-unit Differences in Adiposity Measures among Pregnant Women (n=765) at Enrollment in the PAIR Study, Gaibandha District, Bangladesh, 2018-2019**

Unadjusted models contained just the anthropometric measure. Adjusted 1 models also included drinking water arsenic, age, gestational age, education and living standards index. Adjusted 2 models further included plasma folate, plasma vitamin B12, and plasma homocysteine. Abbreviations: IQR, interquartile range; iAs%, inorganic arsenic percentage; MMA%, monomethyl arsenic percentage; DMA%, dimethyl arsenic percentage; BMI, body mass index; Subscapular, subscapular skinfold thickness; Triceps, triceps skinfold thickness; MUAC, mid-upper arm circumference; MUAFA, mid-upper arm fat area; MUAMA, mid-upper arm muscle area

|  | Mean Difference (95% Confidence Interval) | | |
| --- | --- | --- | --- |
|  | Unadjusted | Adjusted 1 | Adjusted 2 |
| iAs% |  |  |  |
| BMI | -0.87 (-1.32, -0.43) | -0.85 (-1.29, -0.41) | -0.89 (-1.33, -0.46) |
| Subscapular | -0.82 (-1.30, -0.33) | -0.83 (-1.32, -0.34) | -0.83 (-1.32, -0.34) |
| Triceps | -0.92 (-1.41, -0.43) | -0.94 (-1.43, -0.45) | -0.93 (-1.42, -0.45) |
| MUAC | -0.69 (-1.16, -0.22) | -0.68 (-1.15, -0.20) | -0.76 (-1.23, -0.28) |
| MUAFA | -0.84 (-1.32, -0.36) | -0.86 (-1.35, -0.38) | -0.90 (-1.38, -0.42) |
| MUAMA | -0.28 (-0.73, 0.16) | -0.23 (-0.67, 0.21) | -0.34 (-0.78, 0.10) |
| MMA% |  |  |  |
| BMI | -0.72 (-0.94, -0.50) | -0.85 (-1.08, -0.62) | -0.83 (-1.06, -0.60) |
| Subscapular | -0.60 (-0.85, -0.36) | -0.77 (-1.02, -0.51) | -0.75 (-1.00, -0.49) |
| Triceps | -0.67 (-0.92, -0.42) | -0.82 (-1.08, -0.57) | -0.81 (-1.06, -0.55) |
| MUAC | -0.55 (-0.79, -0.31) | -0.71 (-0.96, -0.47) | -0.70 (-0.95, -0.45) |
| MUAFA | -0.65 (-0.89, -0.40) | -0.82 (-1.07, -0.56) | -0.80 (-1.05, -0.55) |
| MUAMA | -0.28 (-0.51, -0.06) | -0.36 (-0.60, -0.13) | -0.36 (-0.59, -0.12) |
| DMA% |  |  |  |
| BMI | 1.59 ( 1.03, 2.16) | 1.70 ( 1.14, 2.27) | 1.72 ( 1.16, 2.28) |
| Subscapular | 1.42 ( 0.79, 2.05) | 1.59 ( 0.97, 2.22) | 1.58 ( 0.95, 2.21) |
| Triceps | 1.59 ( 0.95, 2.22) | 1.77 ( 1.13, 2.40) | 1.74 ( 1.11, 2.37) |
| MUAC | 1.24 ( 0.64, 1.84) | 1.39 ( 0.78, 2.00) | 1.45 ( 0.85, 2.06) |
| MUAFA | 1.48 ( 0.86, 2.10) | 1.68 ( 1.06, 2.30) | 1.70 ( 1.08, 2.31) |
| MUAMA | 0.57 (-0.01, 1.15) | 0.59 ( 0.02, 1.16) | 0.70 ( 0.13, 1.27) |
| PMI |  |  |  |
| BMI | -3.24 (-6.66, 0.30) | -5.30 (-8.76, -1.70) | -4.71 (-8.15, -1.14) |
| Subscapular | -2.18 (-5.99, 1.77) | -4.45 (-8.33, -0.40) | -4.21 (-8.04, -0.22) |
| Triceps | -2.09 (-5.94, 1.91) | -4.24 (-8.16, -0.15) | -4.09 (-7.95, -0.06) |
| MUAC | -2.27 (-5.92, 1.52) | -4.65 (-8.39, -0.76) | -3.87 (-7.59, 0.01) |
| MUAFA | -2.49 (-6.23, 1.39) | -4.77 (-8.59, -0.78) | -4.28 (-8.07, -0.34) |
| MUAMA | -1.69 (-5.18, 1.93) | -3.25 (-6.77, 0.41) | -2.24 (-5.77, 1.42) |
| SMI |  |  |  |
| BMI | 13.08 ( 9.03, 17.29) | 15.27 (11.08, 19.62) | 15.04 (10.86, 19.38) |
| Subscapular | 11.43 ( 7.00, 16.05) | 14.24 ( 9.59, 19.09) | 13.93 ( 9.31, 18.76) |
| Triceps | 12.29 ( 7.78, 16.98) | 15.10 (10.39, 20.02) | 14.86 (10.17, 19.75) |
| MUAC | 9.85 ( 5.64, 14.23) | 12.52 ( 8.08, 17.14) | 12.54 ( 8.10, 17.16) |
| MUAFA | 11.80 ( 7.41, 16.36) | 14.82 (10.21, 19.62) | 14.65 (10.06, 19.44) |
| MUAMA | 4.81 ( 0.95, 8.83) | 5.92 ( 1.99, 10.01) | 6.09 ( 2.14, 10.20) |

**Table S3. Mutually Adjusted Linear Regression Estimates of Mean Differences in Arsenic Methylation Measures per IQR-unit Differences in Mid-upper Arm Fat Area or Mid-upper Arm Muscle Area among Pregnant Women (n=765) at Enrollment in the PAIR Study, Gaibandha District, Bangladesh, 2018-2019**

Models included MUAFA, MUAMA, drinking water arsenic, age, gestational age, education, living standards index, plasma folate, plasma vitamin B12, and plasma homocysteine. Abbreviations: IQR, interquartile range; iAs%, inorganic arsenic percentage; MMA%, monomethyl arsenic percentage; DMA%, dimethyl arsenic percentage; MUAFA, mid-upper arm fat area; MUAMA, mid-upper arm muscle area

|  | Mean Difference |
| --- | --- |
|  | (95% Confidence Interval) |
| iAs% |  |
| MUAFA | -0.95 (-1.51, -0.39) |
| MUAMA | 0.10 (-0.41, 0.60) |
| MMA% |  |
| MUAFA | -0.81 (-1.10, -0.52) |
| MUAMA | 0.02 (-0.25, 0.28) |
| DMA% |  |
| MUAFA | 1.76 ( 1.04, 2.48) |
| MUAMA | -0.11 (-0.77, 0.54) |
| PMI |  |
| MUAFA | -4.10 (-8.49, 0.50) |
| MUAMA | -0.33 (-4.49, 4.01) |
| SMI |  |
| MUAFA | 14.99 ( 9.66, 20.58) |
| MUAMA | -0.52 (-4.72, 3.87) |

**Table S4. Beta Regression Estimates of Differences in Log Odds of Arsenic Methylation Proportions per IQR-unit Differences in Anthropometric Measures among Pregnant Women (n=765) at Enrollment in the PAIR Study, Gaibandha District, Bangladesh, 2018-2019**

Dependent variables were scaled to [0,1] by dividing by 100, but iAs%, MMA%, and DMA% labels are used for consistency. Unadjusted models contained just the anthropometric measure. Adjusted 1 models also included drinking water arsenic, age, gestational age, education and living standards index. Adjusted 2 models further included plasma folate, plasma vitamin B12, and plasma homocysteine. Abbreviations: IQR, interquartile range; iAs%, inorganic arsenic percentage; MMA%, monomethyl arsenic percentage; DMA%, dimethyl arsenic percentage; BMI, body mass index; Subscapular, subscapular skinfold thickness; Triceps, triceps skinfold thickness; MUAC, mid-upper arm circumference; MUAFA, mid-upper arm fat area; MUAMA, mid-upper arm muscle area

|  | Difference in Log Odds (95% Confidence Interval) | | |
| --- | --- | --- | --- |
|  | Unadjusted | Adjusted 1 | Adjusted 2 |
| iAs% |  |  |  |
| BMI | -0.07 (-0.11, -0.03) | -0.07 (-0.11, -0.03) | -0.07 (-0.11, -0.03) |
| Subscapular | -0.07 (-0.11, -0.03) | -0.07 (-0.12, -0.03) | -0.07 (-0.11, -0.03) |
| Triceps | -0.08 (-0.12, -0.03) | -0.08 (-0.12, -0.04) | -0.08 (-0.12, -0.04) |
| MUAC | -0.06 (-0.10, -0.02) | -0.06 (-0.10, -0.02) | -0.07 (-0.11, -0.02) |
| MUAFA | -0.07 (-0.11, -0.03) | -0.07 (-0.12, -0.03) | -0.08 (-0.12, -0.03) |
| MUAMA | -0.02 (-0.06, 0.01) | -0.02 (-0.06, 0.02) | -0.03 (-0.07, 0.01) |
| MMA% |  |  |  |
| BMI | -0.1 (-0.13, -0.07) | -0.12 (-0.15, -0.09) | -0.12 (-0.15, -0.08) |
| Subscapular | -0.09 (-0.13, -0.05) | -0.11 (-0.15, -0.08) | -0.11 (-0.15, -0.07) |
| Triceps | -0.1 (-0.13, -0.06) | -0.12 (-0.16, -0.08) | -0.12 (-0.15, -0.08) |
| MUAC | -0.08 (-0.11, -0.04) | -0.10 (-0.14, -0.07) | -0.1 (-0.14, -0.07) |
| MUAFA | -0.09 (-0.13, -0.06) | -0.12 (-0.16, -0.08) | -0.12 (-0.15, -0.08) |
| MUAMA | -0.04 (-0.07, -0.01) | -0.05 (-0.08, -0.02) | -0.05 (-0.08, -0.02) |
| DMA% |  |  |  |
| BMI | 0.09 (0.06, 0.13) | 0.1 (0.07, 0.14) | 0.10 (0.07, 0.14) |
| Subscapular | 0.09 (0.05, 0.13) | 0.1 (0.06, 0.14) | 0.10 (0.06, 0.14) |
| Triceps | 0.09 (0.05, 0.13) | 0.11 (0.07, 0.15) | 0.10 (0.07, 0.14) |
| MUAC | 0.07 (0.04, 0.11) | 0.08 (0.05, 0.12) | 0.09 (0.05, 0.13) |
| MUAFA | 0.09 (0.05, 0.13) | 0.10 (0.06, 0.14) | 0.10 (0.06, 0.14) |
| MUAMA | 0.03 (0.00, 0.07) | 0.04 (0.00, 0.07) | 0.04 (0.01, 0.08) |

**Table S5. Dirichlet Regression Estimates of Differences in Log Odds of Arsenic Methylation Proportions per IQR-unit Differences in Anthropometric Measures among Pregnant Women (n=765) at Enrollment in the PAIR Study, Gaibandha District, Bangladesh, 2018-2019**

Dirichlet regression omits one dependent variable (here, iAs%), which is estimated implicitly. Dependent variables were scaled to [0,1] by dividing by 100, but iAs%, MMA%, and DMA% labels are used for consistency. Unadjusted models contained just the anthropometric measure. Adjusted 1 models also included drinking water arsenic, age, gestational age, education and living standards index. Adjusted 2 models further included plasma folate, plasma vitamin B12, and plasma homocysteine. Abbreviations: IQR, interquartile range; iAs%, inorganic arsenic percentage; MMA%, monomethyl arsenic percentage; DMA%, dimethyl arsenic percentage; BMI, body mass index; MUAC, mid-upper arm circumference; MUAFA, mid-upper arm fat area; MUAMA, mid-upper arm muscle area

|  | Difference in Log Odds (95% Confidence Interval) | | |
| --- | --- | --- | --- |
|  | Unadjusted | Adjusted 1 | Adjusted 2 |
| BMI |  |  |  |
| iAs% | (Omitted) | (Omitted) | (Omitted) |
| MMA% | -0.02 (-0.08, 0.03) | -0.04 (-0.10, 0.01) | -0.04 (-0.09, 0.01) |
| DMA% | 0.08 (0.05, 0.12) | 0.08 (0.05, 0.12) | 0.09 (0.05, 0.12) |
| Subscapular Skinfold |  |  |  |
| iAs% | (Omitted) | (Omitted) | (Omitted) |
| MMA% | -0.01 (-0.07, 0.04) | -0.04 (-0.10, 0.02) | -0.04 (-0.09, 0.02) |
| DMA% | 0.08 (0.04, 0.12) | 0.08 (0.05, 0.12) | 0.08 (0.04, 0.12) |
| Triceps Skinfold |  |  |  |
| iAs% | (Omitted) | (Omitted) | (Omitted) |
| MMA% | -0.02 (-0.07, 0.04) | -0.04 (-0.09, 0.02) | -0.04 (-0.09, 0.02) |
| DMA% | 0.09 (0.05, 0.13) | 0.09 (0.05, 0.13) | 0.09 (0.05, 0.13) |
| MUAC |  |  |  |
| iAs% | (Omitted) | (Omitted) | (Omitted) |
| MMA% | -0.02 (-0.07, 0.04) | -0.04 (-0.09, 0.02) | -0.03 (-0.09, 0.02) |
| DMA% | 0.07 (0.03, 0.10) | 0.07 (0.03, 0.10) | 0.08 (0.04, 0.11) |
| MUAFA |  |  |  |
| iAs% | (Omitted) | (Omitted) | (Omitted) |
| MMA% | -0.02 (-0.07, 0.04) | -0.04 (-0.10, 0.02) | -0.04 (-0.09, 0.02) |
| DMA% | 0.08 (0.04, 0.12) | 0.09 (0.05, 0.12) | 0.09 (0.05, 0.13) |
| MUAMA |  |  |  |
| iAs% | (Omitted) | (Omitted) | (Omitted) |
| MMA% | -0.01 (-0.06, 0.04) | -0.03 (-0.08, 0.02) | -0.02 (-0.07, 0.03) |
| DMA% | 0.03 (-0.01, 0.06) | 0.02 (-0.01, 0.06) | 0.04 (0.00, 0.07) |

**Table S6. Mutually Adjusted Beta Regression Estimates of Differences in Log Odds of Arsenic Methylation Proportions per IQR-unit Differences in Mid-upper Arm Fat Area or Mid-upper Arm Muscle Area among Pregnant Women (n=765) at Enrollment in the PAIR Study, Gaibandha District, Bangladesh, 2018-2019**

Dependent variables were scaled to [0,1] by dividing by 100, but iAs%, MMA%, and DMA% labels are used for consistency. Models included MUAFA, MUAMA, drinking water arsenic, age, gestational age, education, living standards index, plasma folate, plasma vitamin B12, and plasma homocysteine. Abbreviations: IQR, interquartile range; iAs%, inorganic arsenic percentage; MMA%, monomethyl arsenic percentage; DMA%, dimethyl arsenic percentage; MUAFA, mid-upper arm fat area; MUAMA, mid-upper arm muscle area

|  | Difference in Log Odds |
| --- | --- |
|  | (95% Confidence Interval) |
| iAs% |  |
| MUAFA | -0.08 (-0.13, -0.03) |
| MUAMA | 0.00 (-0.04, 0.05) |
| MMA% |  |
| MUAFA | -0.12 (-0.16, -0.08) |
| MUAMA | 0.00 (-0.04, 0.04) |
| DMA% |  |
| MUAFA | 0.10 ( 0.06, 0.15) |
| MUAMA | 0.00 (-0.04, 0.04) |

**Table S7. Mutually Adjusted Dirichlet Regression Estimates of Differences in Log Odds of Arsenic Methylation Proportions per IQR-unit Differences in Mid-upper Arm Fat Area or Mid-upper Arm Muscle Area among Pregnant Women (n=765) at Enrollment in the PAIR Study, Gaibandha District, Bangladesh, 2018-2019**

Dirichlet regression omits one dependent variable (here, iAs%), which is estimated implicitly. Dependent variables were scaled to [0,1] by dividing by 100, but iAs%, MMA%, and DMA% labels are used for consistency. Models included MUAFA, MUAMA, drinking water arsenic, age, gestational age, education, living standards index, plasma folate, plasma vitamin B12, and plasma homocysteine. Abbreviations: IQR, interquartile range; iAs%, inorganic arsenic percentage; MMA%, monomethyl arsenic percentage; DMA%, dimethyl arsenic percentage; MUAFA, mid-upper arm fat area; MUAMA, mid-upper arm muscle area

|  | Difference in Log Odds (95% Confidence Interval) | |
| --- | --- | --- |
|  | MUAFA | MUAMA |
| iAs% | (Omitted) | (Omitted) |
| MMA% | -0.04 (-0.10, 0.03) | 0.00 (-0.06, 0.06) |
| DMA% | 0.09 ( 0.05, 0.13) | 0.00 (-0.04, 0.03) |

**Figure S1. Histograms of Arsenic Methylation Measure Z-Scores on Linear and Natural Log Scales among Pregnant Women (n=765) at Enrollment in the PAIR Study, Gaibandha District, Bangladesh, 2018-2019**

Abbreviations: iAs%, inorganic arsenic percentage; MMA%, monomethyl arsenic percentage; DMA%, dimethyl arsenic percentage; PMI, primary methylation index (MMA/iAs); SMI, secondary methylation index (DMA/MMA)


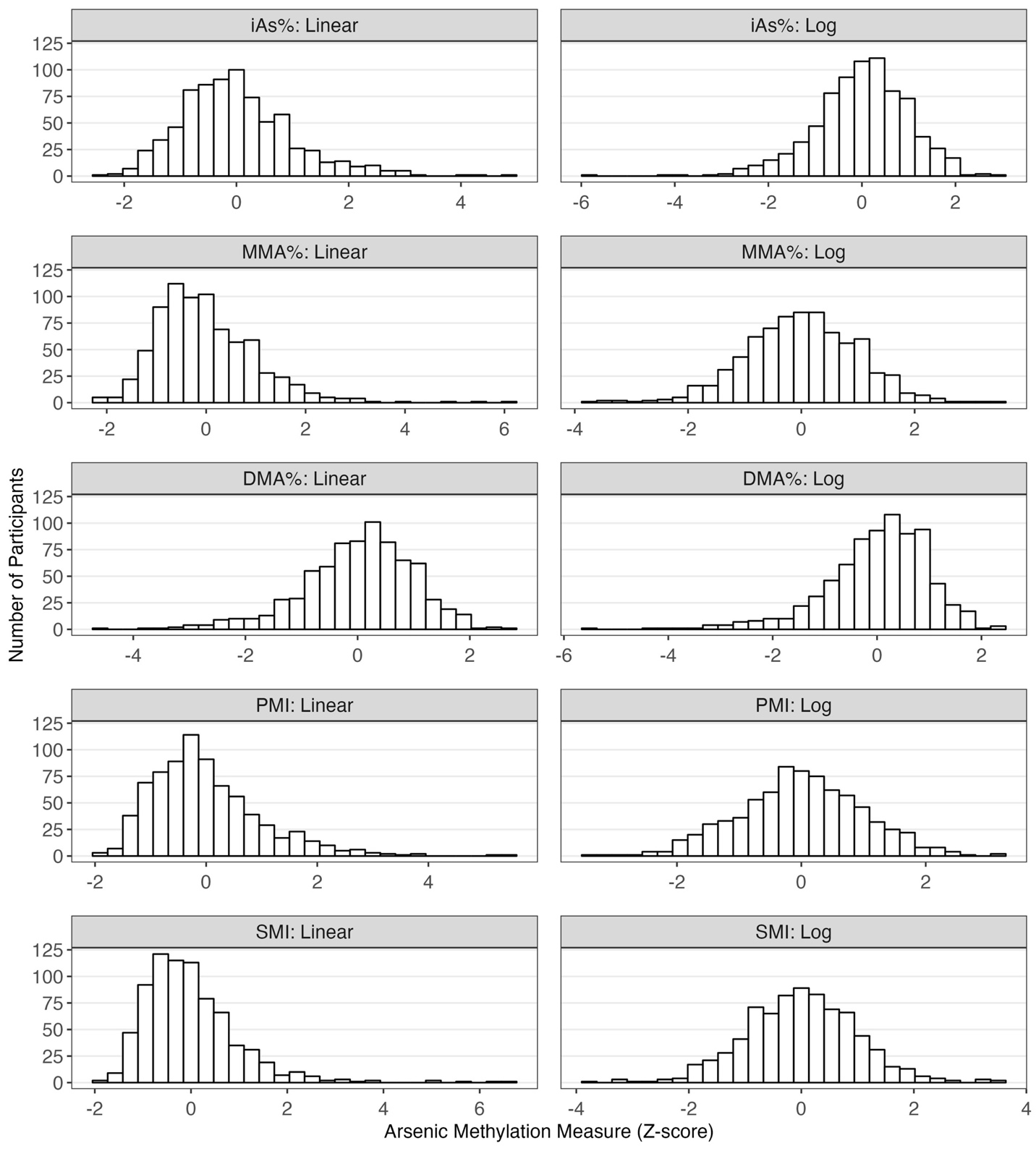


**Figure S2. Scatter Plots of Arsenic Methylation Measures by Body Mass Index (BMI) among Pregnant Women (n=765) at Enrollment in the PAIR Study, Gaibandha District, Bangladesh, 2018-2019**

Red solid curve is a locally estimated scatterplot smoother (LOESS) and blue dashed curve is a linear smoother. Abbreviations: BMI, body mass index; iAs%, inorganic arsenic percentage; MMA%, monomethyl arsenic percentage; DMA%, dimethyl arsenic percentage; PMI, primary methylation index (MMA/iAs); SMI, secondary methylation index (DMA/MMA)


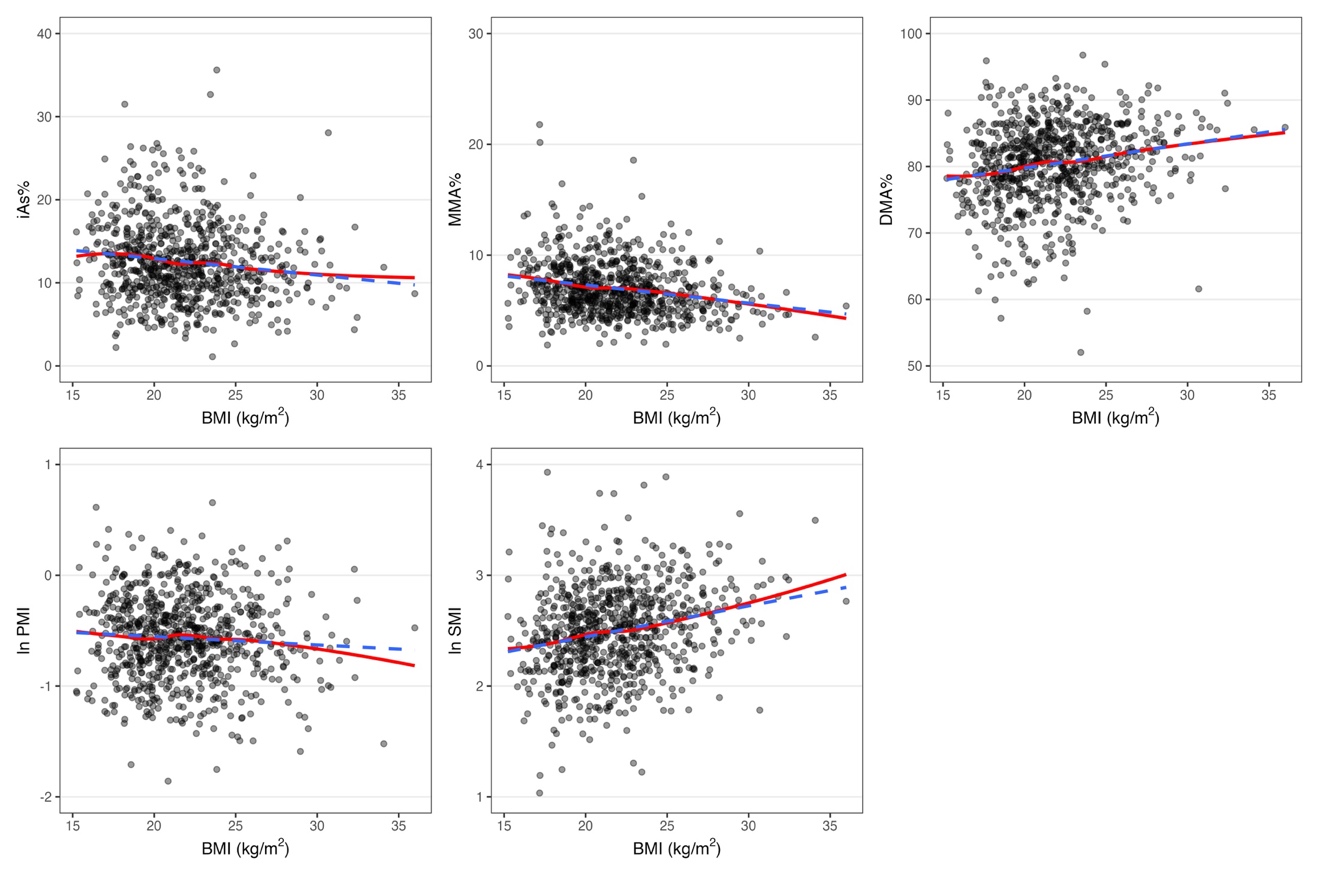


**Figure S3. Scatter Plots of Arsenic Methylation Measures by Subscapular Skinfold Thickness among Pregnant Women (n=765) at Enrollment in the PAIR Study, Gaibandha District, Bangladesh, 2018-2019**

Red solid curve is a locally estimated scatterplot smoother (LOESS) and blue dashed curve is a linear smoother. Abbreviations: iAs%, inorganic arsenic percentage; MMA%, monomethyl arsenic percentage; DMA%, dimethyl arsenic percentage; PMI, primary methylation index (MMA/iAs); SMI, secondary methylation index (DMA/MMA)


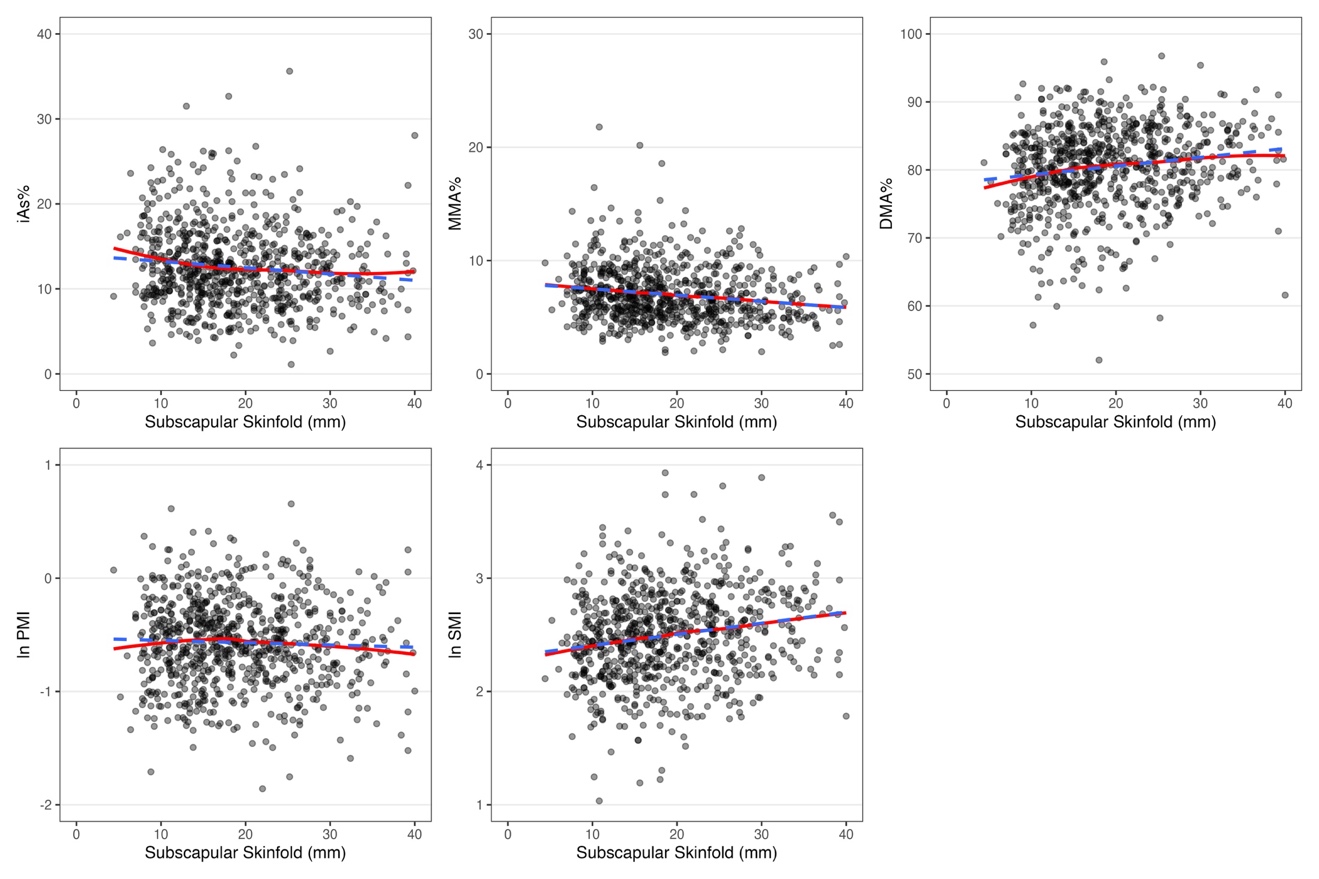


**Figure S4. Scatter Plots of Arsenic Methylation Measures by Triceps Skinfold Thickness among Pregnant Women (n=765) at Enrollment in the PAIR Study, Gaibandha District, Bangladesh, 2018-2019**

Red solid curve is a locally estimated scatterplot smoother (LOESS) and blue dashed curve is a linear smoother. Abbreviations: iAs%, inorganic arsenic percentage; MMA%, monomethyl arsenic percentage; DMA%, dimethyl arsenic percentage; PMI, primary methylation index (MMA/iAs); SMI, secondary methylation index (DMA/MMA)


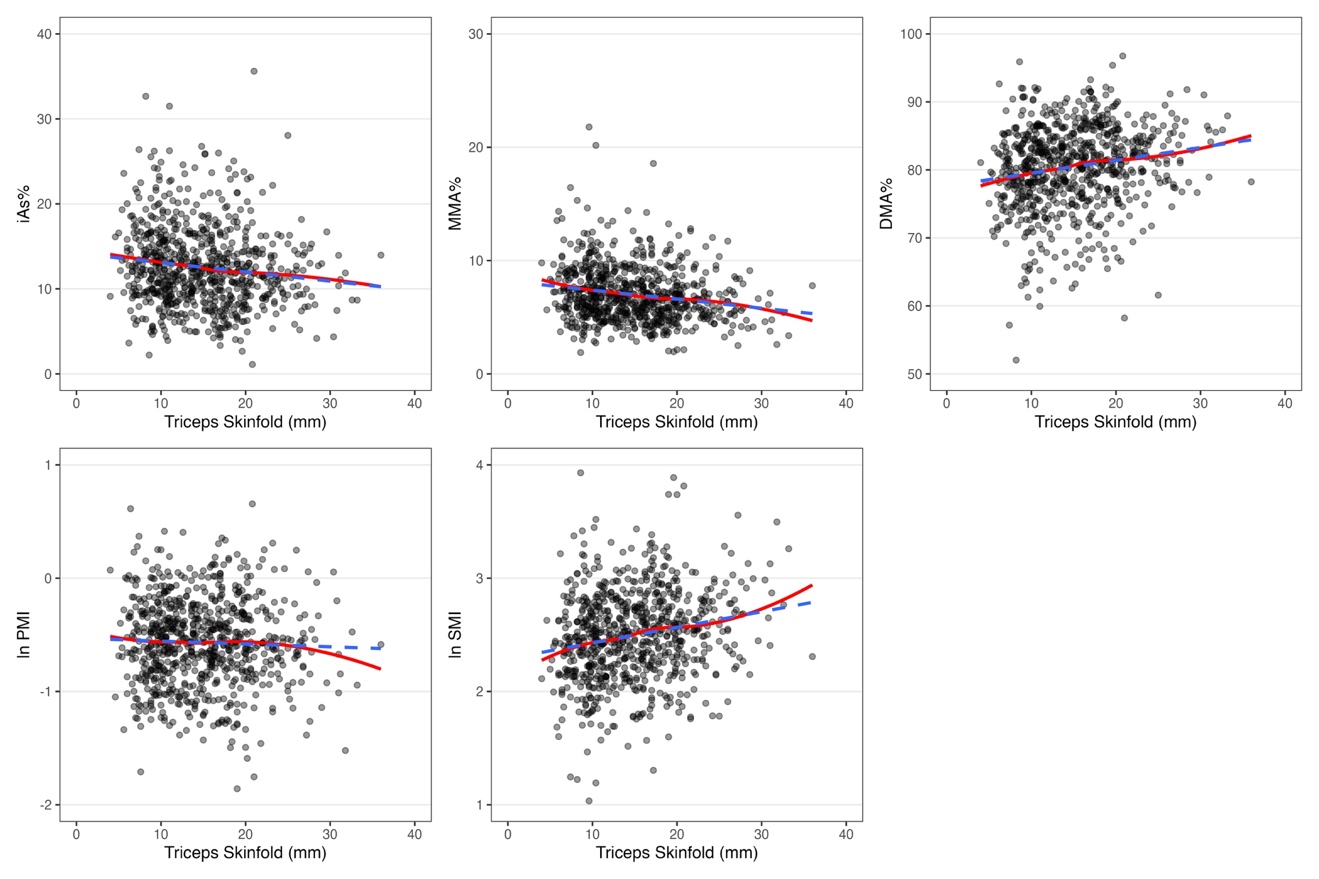


**Figure S5. Scatter Plots of Arsenic Methylation Measures by Mid-upper Arm Circumference (MUAC) among Pregnant Women (n=765) at Enrollment in the PAIR Study, Gaibandha District, Bangladesh, 2018-2019**

Red solid curve is a locally estimated scatterplot smoother (LOESS) and blue dashed curve is a linear smoother. Abbreviations: MUAC, mid-upper arm circumference; iAs%, inorganic arsenic percentage; MMA%, monomethyl arsenic percentage; DMA%, dimethyl arsenic percentage; PMI, primary methylation index (MMA/iAs); SMI, secondary methylation index (DMA/MMA)


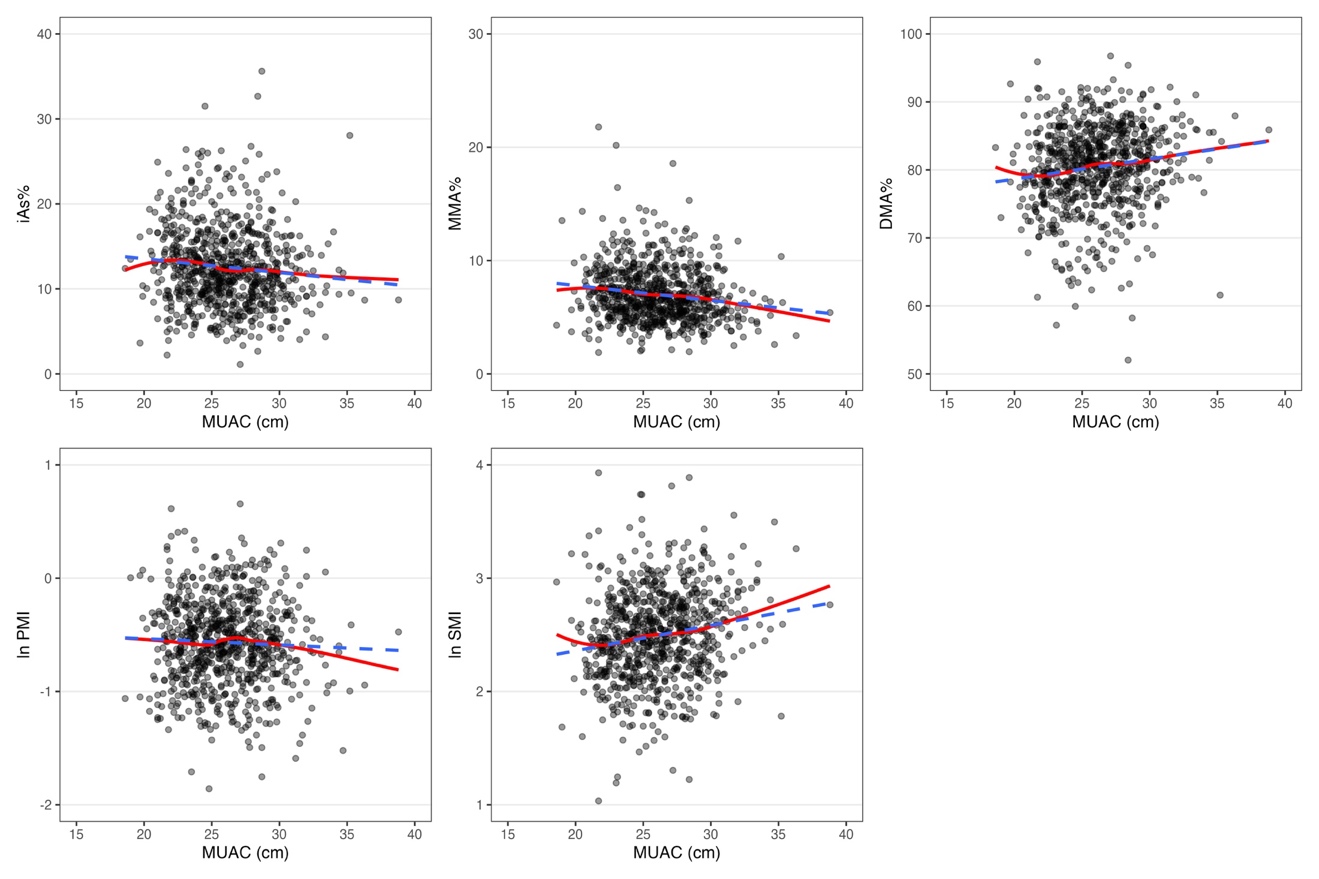


**Figure S6. Scatter Plots of Arsenic Methylation Measures by Mid-upper Arm Fat Area (MUAFA) among Pregnant Women (n=765) at Enrollment in the PAIR Study, Gaibandha District, Bangladesh, 2018-2019**

Red solid curve is a locally estimated scatterplot smoother (LOESS) and blue dashed curve is a linear smoother. Abbreviations: MUAFA, mid-upper arm fat area; iAs%, inorganic arsenic percentage; MMA%, monomethyl arsenic percentage; DMA%, dimethyl arsenic percentage; PMI, primary methylation index (MMA/iAs); SMI, secondary methylation index (DMA/MMA)


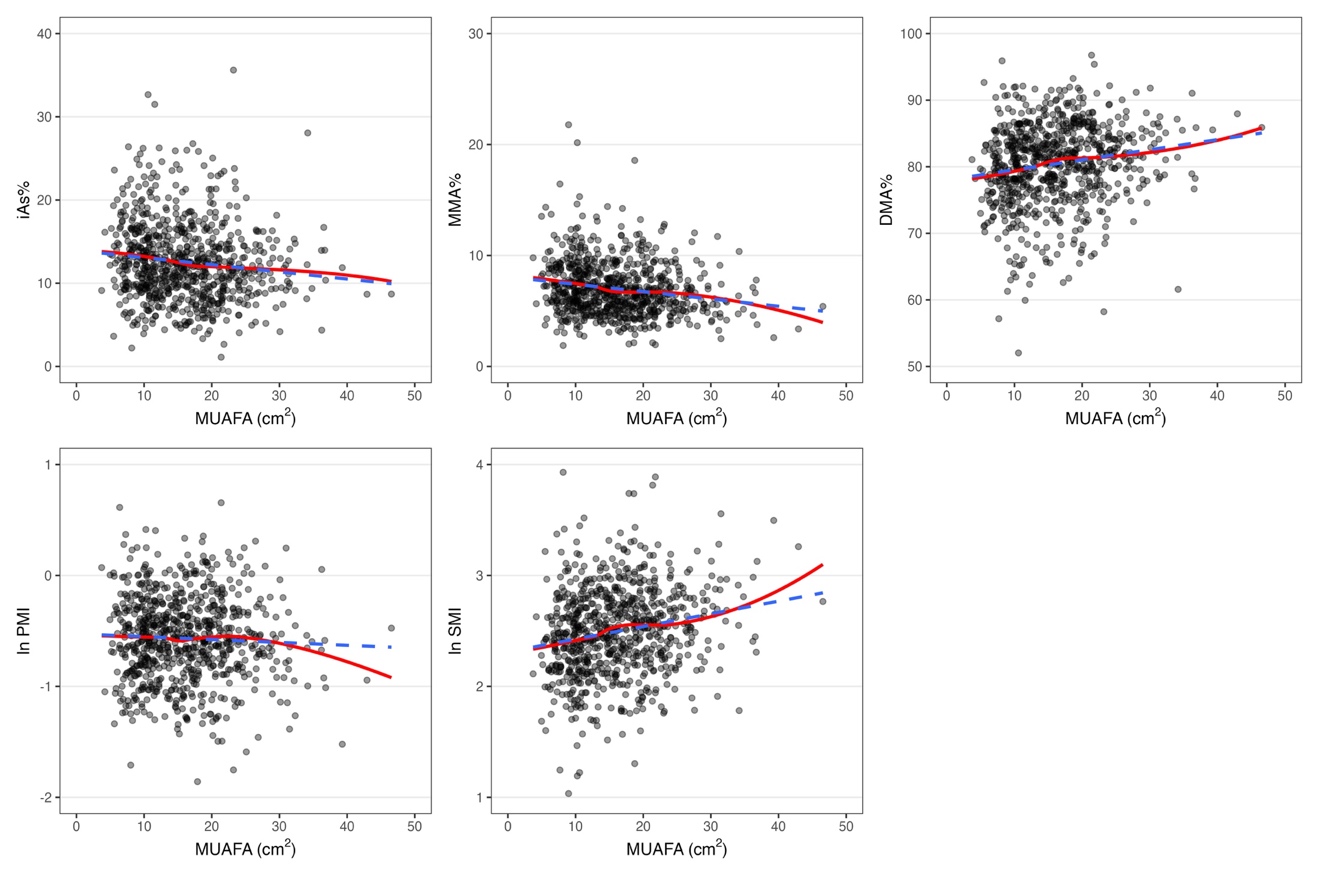


**Figure S7. Scatter Plots of Arsenic Methylation Measures by Mid-upper Arm Muscle Area (MUAMA) among Pregnant Women (n=765) at Enrollment in the PAIR Study, Gaibandha District, Bangladesh, 2018-2019**

Red solid curve is a locally estimated scatterplot smoother (LOESS) and blue dashed curve is a linear smoother. Abbreviations: MUAMA, mid-upper arm muscle area; iAs%, inorganic arsenic percentage; MMA%, monomethyl arsenic percentage; DMA%, dimethyl arsenic percentage; PMI, primary methylation index (MMA/iAs); SMI, secondary methylation index (DMA/MMA)


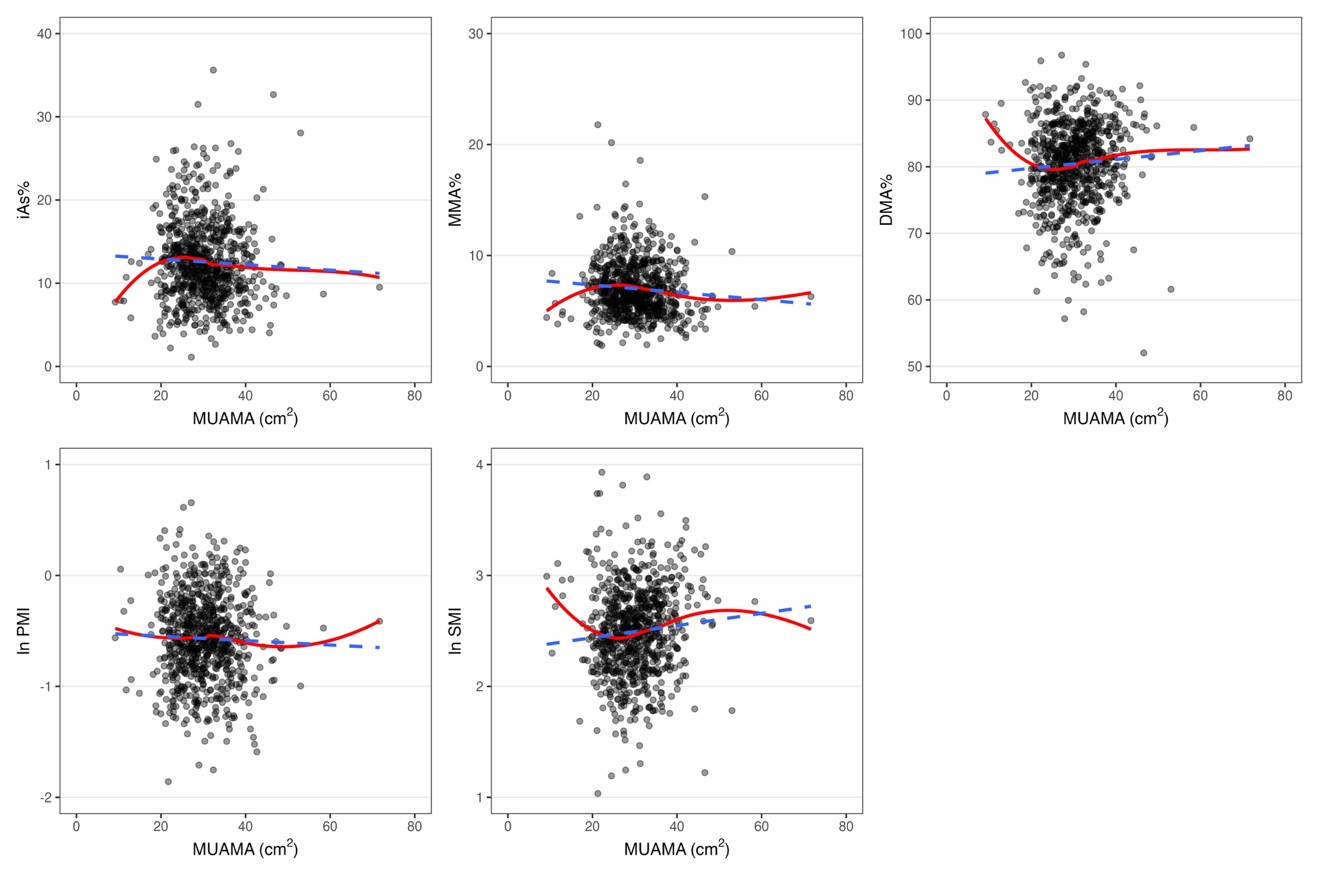


**Figure S8. Beta Regression Estimates of Differences in Log Odds of Arsenic Methylation Proportions per IQR-unit Differences in Anthropometric Measures among Pregnant Women (n=765) at Enrollment in the PAIR Study, Gaibandha District, Bangladesh, 2018-2019**

Dependent variables were scaled to [0,1] by dividing by 100, but iAs%, MMA%, and DMA% labels are used for consistency. Unadjusted models contained just the adiposity measure. Adjusted 1 models also included drinking water arsenic, age, gestational age, education and living standards index. Adjusted 2 models further included plasma folate, plasma vitamin B12, and plasma homocysteine. Abbreviations: IQR, interquartile range; iAs%, inorganic arsenic percentage; MMA%, monomethyl arsenic percentage; DMA%, dimethyl arsenic percentage; BMI, body mass index; Subscapular, subscapular skinfold thickness; Triceps, triceps skinfold thickness; MUAC, mid-upper arm circumference; MUAFA, mid-upper arm fat area; MUAMA, mid-upper arm muscle area


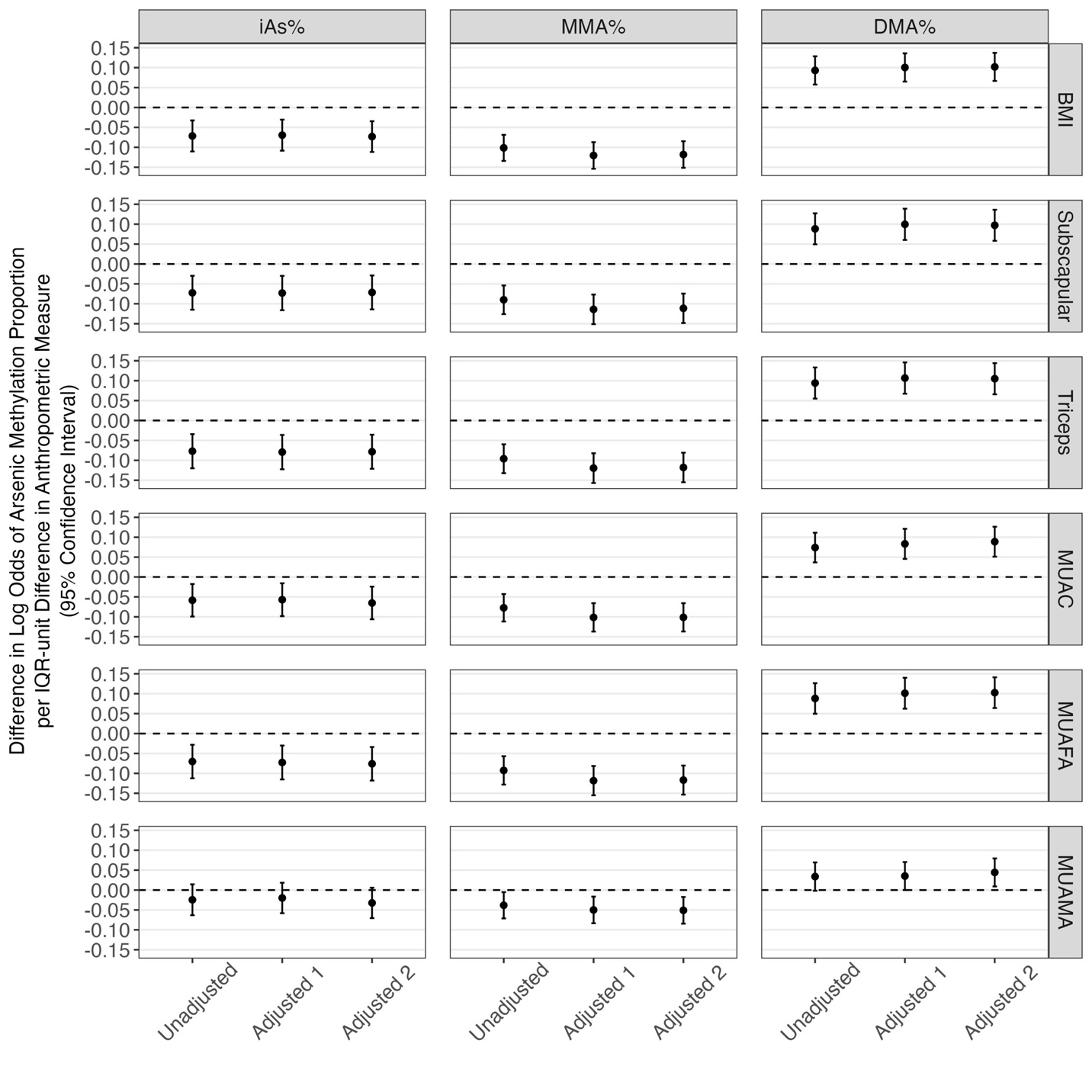


**Figure S9. Dirichlet Regression Estimates of Differences in Log Odds of Arsenic Methylation Proportions per IQR-unit Differences in Anthropometric Measures among Pregnant Women (n=765) at Enrollment in the PAIR Study, Gaibandha District, Bangladesh, 2018-2019**

Dirichlet regression omits one dependent variable (here, iAs%), which is estimated implicitly. Dependent variables were scaled to [0,1] by dividing by 100, but MMA% and DMA% labels are used for consistency. Unadjusted models contained just the adiposity measure. Adjusted 1 models also included drinking water arsenic, age, gestational age, education and living standards index. Adjusted 2 models further included plasma folate, plasma vitamin B12, and plasma homocysteine. Abbreviations: IQR, interquartile range; iAs%, inorganic arsenic percentage; MMA%, monomethyl arsenic percentage; DMA%, dimethyl arsenic percentage; BMI, body mass index; Subscapular, subscapular skinfold thickness; Triceps, triceps skinfold thickness; MUAC, mid-upper arm circumference; MUAFA, mid-upper arm fat area; MUAMA, mid-upper arm muscle area


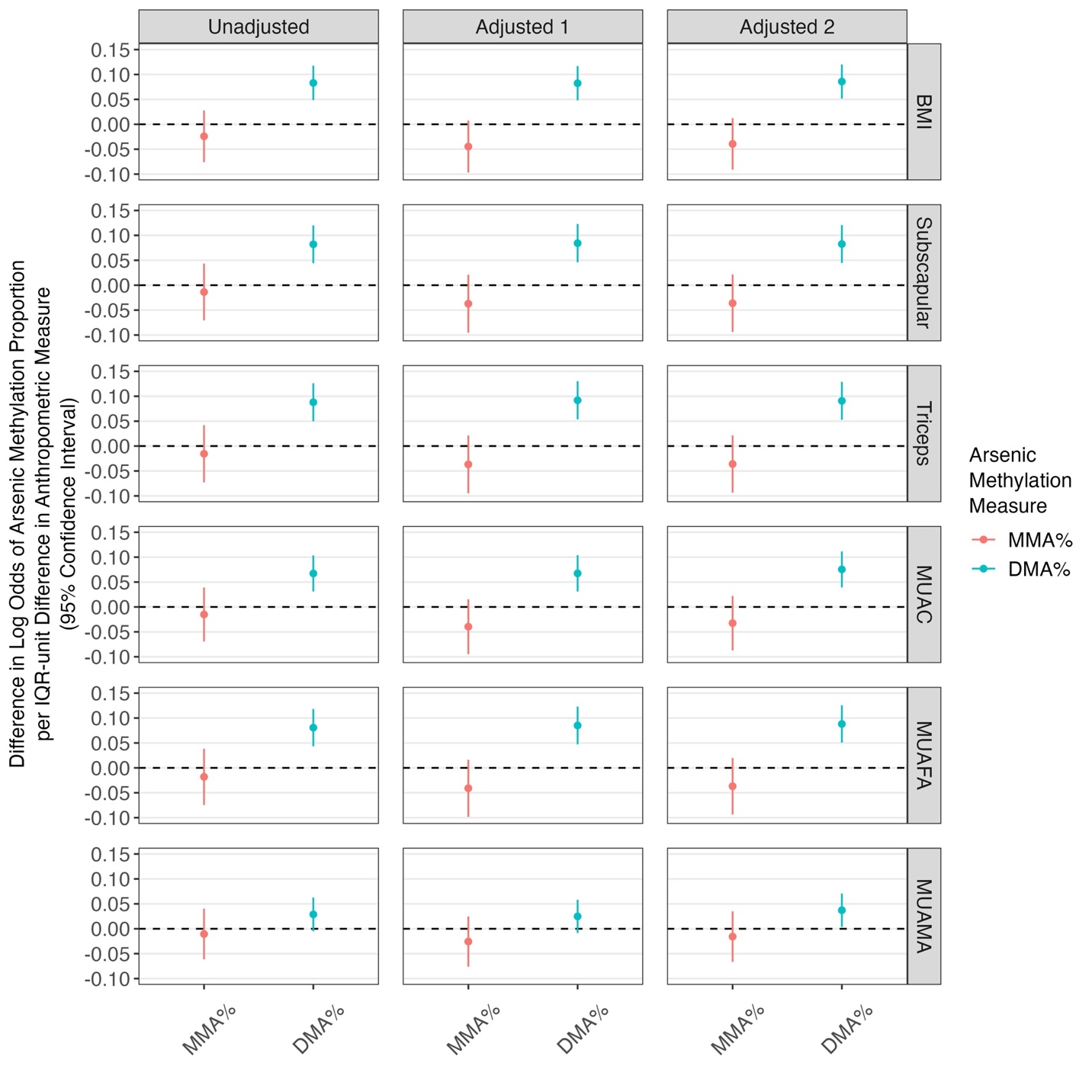

Supplement: Multimedia component 1 [file mmc1.docx]
